# Regenerating Locus Coeruleus‐Norepinephrine (LC‐NE) Function: A Novel Approach for Neurodegenerative Diseases

**DOI:** 10.1111/cpr.13807

**Published:** 2025-01-28

**Authors:** Yana Yang, Yunlong Tao

**Affiliations:** ^1^ State Key Laboratory of Pharmaceutical Biotechnology, School of Life Sciences, Chemistry and Biomedicine Innovation Center (ChemBIC) Nanjing University Nanjing China

**Keywords:** locus coeruleus, neurodegenerative diseases, norepinephrine neurons, pluripotent stem cells, psychiatric diseases

## Abstract

Pathological changes in the locus coeruleus‐norepinephrine (LC‐NE) neurons, the major source of norepinephrine (NE, also known as noradrenaline) in the brain, are evident during the early stages of neurodegenerative diseases (ND). Research on both human and animal models have highlighted the therapeutic potential of targeting the LC‐NE system to mitigate the progression of ND and alleviate associated psychiatric symptoms. However, the early and widespread degeneration of the LC‐NE system presents a significant challenge for direct intervention in ND. Recent advances in regenerative cell therapy offer promising new strategies for ND treatment. The regeneration of LC‐NE from pluripotent stem cells (PSCs) could significantly broaden the scope of LC‐NE‐based therapies for ND. In this review, we delve into the fundamental background and physiological functions of LC‐NE. Additionally, we systematically examine the evidence and role of the LC‐NE system in the neuropathology of ND and psychiatric diseases over recent years. Notably, we focus on the significance of PSCs‐derived LC‐NE and its potential impact on ND therapy. A deeper understanding and further investigation into the regeneration of LC‐NE function could pave the way for practical and effective treatments for ND.

## Graphical abstract

The regeneration of LC‐NE from pluripotent stem cells (PSCs) could significantly mitigate the progression of Neurodegenerative diseases and alleviate associated psychiatric disease symptoms, providing a new approach to the treatment of neurological diseases.

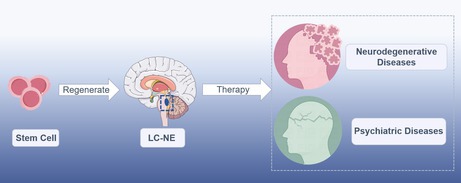

## 1 Introduction

The LC, the main NE nucleus in the brain, plays a pivotal role in managing alert states, attention, stress responses and behavioural arousal [1]. In addition to these functions, NE released by LC terminals exerts multiple effects. Studies have highlighted the evident pathological changes in the LC‐NE system during the early stages of ND, and that LC‐NE disorders are closely associated with the onset and progression of psychiatric symptoms [2]. Reducing LC neuron damage or enhancing LC neuron activity and NE activity may offer therapeutic benefits in slowing the progression of ND and associated psychiatric conditions. Unfortunately, the early and extensive degeneration of the LC‐NE system in many ND cases makes them as direct target very challenging. Given these complexities, it becomes crucial to restore LC‐NE function by regenerating LC‐NE neurons derived from PSCs or through trans‐differentiation of brain cells.

ND, including Alzheimer's disease (AD), Parkinson's disease (PD) and Multiple sclerosis (MS), are characterised by progressive loss of selectively vulnerable neuronal populations and myelin sheath, leading to behavioural and cognitive dysfunction that adversely affects the quality of life. However, the vast majority of drugs have huge limitations in crossing the blood–brain barrier (BBB), which currently can only alleviate rather than cure [3]. Stem cell therapy has emerged as a powerful strategy for addressing these challenging conditions [4]. If LC‐NE regeneration can be achieved through neural differentiation from PSCs, it could revolutionise therapeutic strategies for ND. Recent studies have begun to overcome many of these challenges, enabling LC‐NE regeneration through PSCs.

This review provides an overview of the basic physiological functions of LC‐NE and systematically examines its dysregulation in ND and psychiatric disorders. Additionally, we discuss recent advances in stem cell therapy for ND, with a particular focus on the potential of PSC‐derived LC‐NE to transform the treatment landscape.

## 2 The Locus Coeruleus‐Norepinephrine Neuron

**FIGURE 1:**
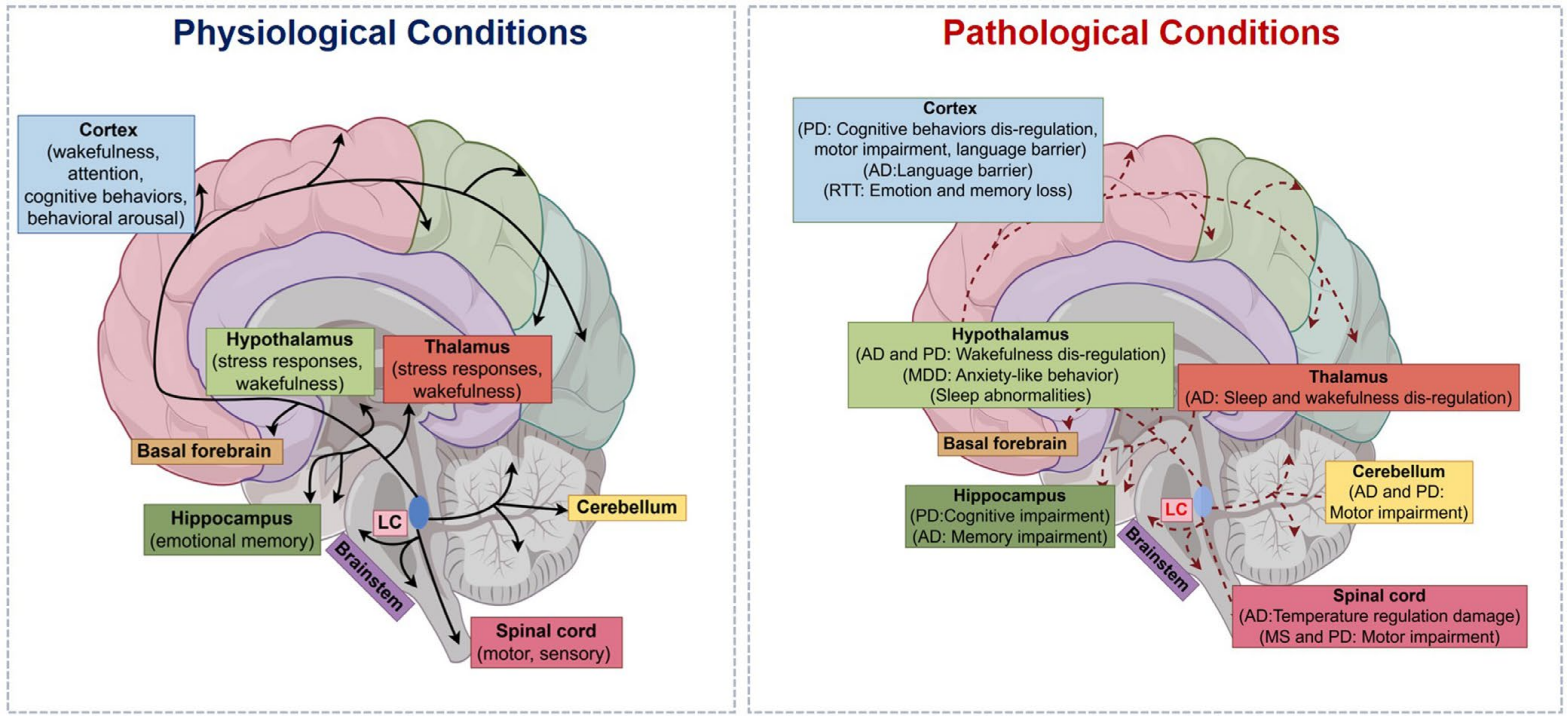
Projection area of LC and its function. LC, a small nucleus located at the base of the fourth ventricle in the brainstem's posterior region and the Pontine's anterior dorsal segment, is the main source of NE. LC projects to multiple areas including cortex, hippocampus, hypothalamus, basal forebrain, thalamus, brainstem, basal ganglia, cerebellum and spinal cord. In physiological situation, these pathways are associated with the normal functioning of wakefulness, cognition, movement, stress response and other functions (black arrows). However, LC‐NE dysfunction may lead to abnormalities in the above pathways, contributing to the emergence of psychiatric diseases and ND, including AD, PD and MS (red arrows). The figure by Figdraw.

LC, located at the base of the fourth ventricle in the brainstem's posterior region and the Pontine's anterior dorsal segment, is primarily composed of NE neurons [5]. Despite its modest neuronal population—ranging from 10,000 to 30,000 in non‐human primates and 20,000 to 50,000 in humans [6, 7], the LC's axons exhibit extensive branching and reach numerous brain areas, including the cortex, hippocampus, brainstem, basal ganglia and spinal cord. This vast network allows the LC to play a significant role in regulating functions across these regions [8, 9, 10] (Figure 1). Upon release, NE from LC neurons bind to adrenergic receptors, initiating a cascade of downstream pathways that influence neurotransmitter release, neuroinflammation and growth factor expression, thereby contributing to various brain functions such as wakefulness, learning, memory and a range of cognitive behaviours [11, 12, 13]. LC‐NE is strongly associated with alert states and is believed to be part of a broader network that includes neurons in the brainstem, midbrain, thalamus and hypothalamus [1]. It manifests that although LC‐NE remain silent during rapid eye movement (REM) sleep and show low levels of activity during non‐REM sleep, they are most active when awake [14]. LC‐NE activity exhibits different spatiotemporal dynamics during learning behaviour, which in turn implements two functions: facilitating task execution and encoding reinforcement to improve performance accuracy [15]. Additionally, the LC‐NE system is pivotal in managing attention, stress responses, emotional memory and behavioural arousal [16].

Historically, the LC has long been considered a homogeneous nucleus because of its common embryonic origin and the production of NE by its neurons [17]. However, accumulating evidence reveals considerable diversity in the LC's architecture and functionality [18, 19]. The molecule and function of mouse NE in LC vary by sex [20]. Recent studies, using advanced techniques like spatially resolved transcriptomics and single‐nucleus RNA sequencing, have further dissected the molecular landscape of the human LC region. These studies identified distinct populations of NE neurons and serotonin (5‐HT) neurons, precisely localising them within the LC and its surrounding regions. Novel sets of significantly differentially expressed (DE) genes, including 13 protein‐coding mitochondrial genes, have been discovered, with gene expression varying spatially across the neuroanatomy [21]. These compelling findings underscore the remarkable heterogeneity of the LC.

Two primary types of LC‐NE have been identified: medium‐sized neurons (35–45 μm in body diameter) with multiple dendrites and a large axon, and smaller NE neurons (about 15–20 μm in body diameter) with a spindle‐like soma [22]. While both types are widely distributed throughout the LC, their distribution is skewed. More spindle cells are found in the dorsal LC, and more multipolar cells are located in the ventral LC [23]. In addition to differences in morphology and location, they exhibit selective projections to distinct brain regions. Medium‐sized neurons primarily project to spinal cord and cerebellum, while others target regions such as the hypothalamus, hippocampus and cortex [9]. LC neurons express several neuropeptides, including galanin (Gal), neuropeptide Y (NPY), with Gal co‐expressed in up to 80% of LC neurons [24, 25]. Gal and NE are mainly co‐expressed in the dorsal and central LC sub‐regions, while NPY is co‐expressed predominantly in the dorsal part of LC [24]. LC neurons also contain a variety of neurotransmitter receptors, including several subtypes of adrenergic receptors [26], nicotinic acetylcholine receptors (nAChR) [27], gamma‐aminobutyric acid type a receptor (GABA) receptors [28], Orexin/hypocretin [29], Opioid receptors [30]. The differential expression of these receptors on LC neurons results in varied responses to similar events [25]. For example, nAChR is highly expressed in the LC, but its distribution between medium‐sized and smaller NE neurons results in different response intensities, with nicotine promoting LC neuron depolarization and increasing their firing rate [27, 31].

**FIGURE 2:**
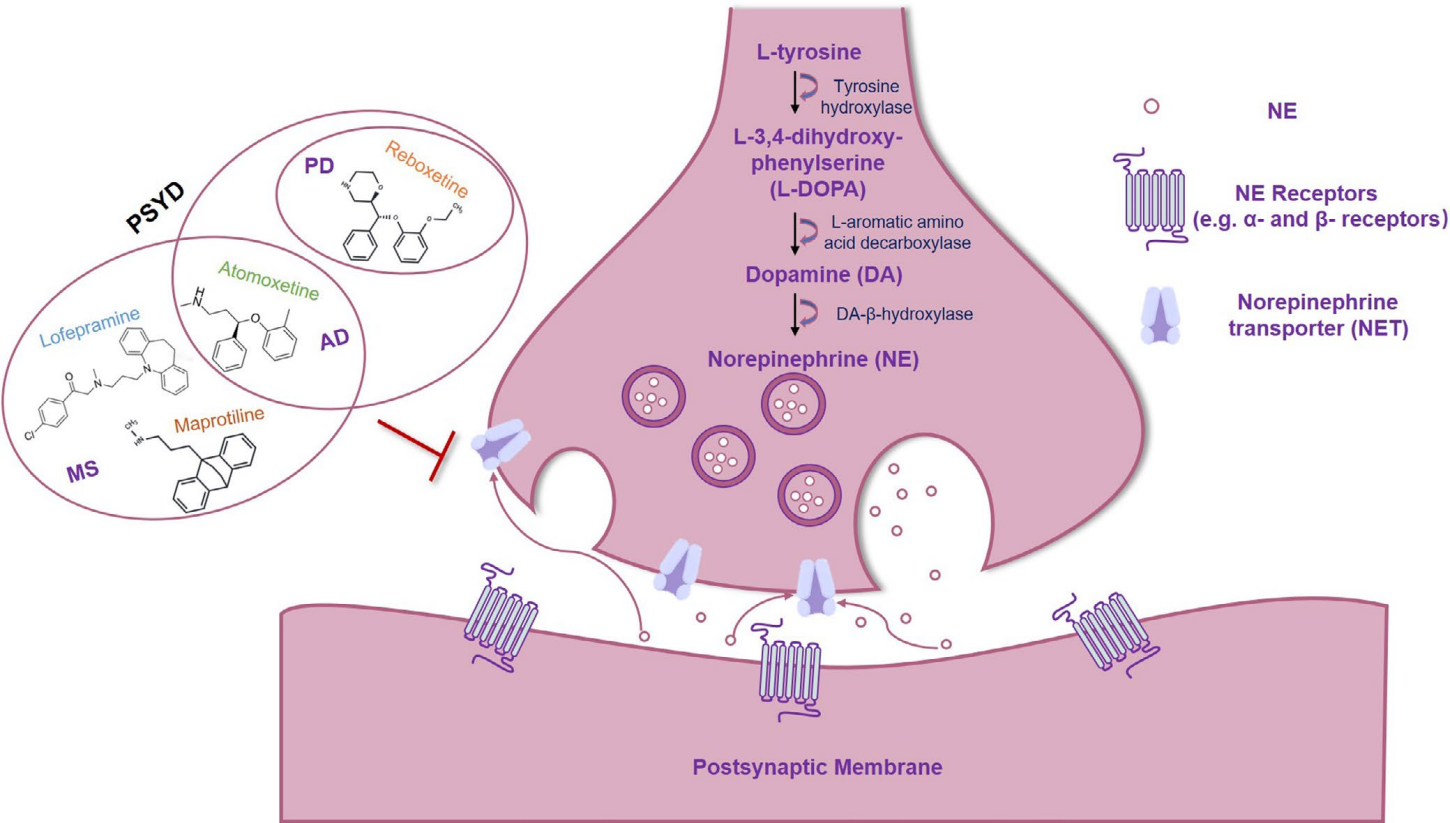
Biosynthetic and metabolic pathways of NE and the drugs targeting NE to treat ND and psychiatric disorders. L‐tyrosine undergoes a series of enzymatic reactions in LC to produce NE, which is released from the presynaptic membrane into the synaptic cleft and binds the NE receptor, thus initiating a series of signal transduction processes. The norepinephrine transporter (NET) located on the presynaptic membrane can reuptake NE back into the presynaptic neurons, and terminate the neural signal transmission, thus maintaining the NE level of balance in the nervous system, which enables it to perform normal physiological functions. A variety of small molecule drugs, such as Lofepramine, Atomoxetine, Maprotiline, Reboxetine, treat ND and psychiatric disorders (PSYD) by inhibiting NET to increase the concentration of NE in the synaptic gap, which in turn enhances the signalling of NE.

NE is a pivotal neurotransmitter synthesised from L‐phenylalanine and L‐tyrosine. These precursors are part of a shared multi‐enzyme pathway that also leads to the production of dopamine (DA). The synthesis begins with tyrosine hydroxylase (TH), which catalyses the conversion of L‐phenylalanine and L‐tyrosine to L‐3, 4‐dihydroxyphenylalanine (L‐DOPA). L‐DOPA is then converted to DA by L‐aromatic amino acid decarboxylase. Subsequently, DA is catalysed by DA‐β‐hydroxylase to form NE. Only neurons expressing DA‐β‐hydroxylase are capable of NE neurotransmission, which in turn acts on G‐coupled α‐ and β‐adrenoreceptors in neurons, glial cells and other cell types [32, 33, 34] (Figure 2). In the brain, NE is primarily produced by norepinephrine neurons in the LC, which project to nearly all brain regions [14, 35, 36]. However, the density of NE innervation varies across the cerebral cortex, with the most extensive innervation observed in the somatosensory and motor cortices, as well as the prefrontal and parietal cortices [37, 38, 39]. The presence of NE is crucial for brain development, and its absence can lead to significant developmental and behavioural abnormalities. Specifically, the LC is the sole source of cortical NE, and early depletion of NE results in atypical cortical development [40, 41]. Furthermore, NE deficiency is linked to delayed cerebellar development and leads to programming reactivity disorders of other neurotransmitter systems in the brain [42]. Studies on DA‐β‐hydroxylase knockout mice reveal that chronic NE loss leads to reduced DA levels, increasing vulnerability to substances like cocaine [43]. Recent studies indicate that NE depletion affects memory retrieval and reversal learning differently in male and female rats [44]. Additionally, dysregulated LC‐NE activity is associated with anxiety‐like behaviours, potentially contributing to the early anxious symptoms of depression and AD [45, 46]. The dysfunction of the LC‐NE system has been implicated in a range of behavioural, neuropsychiatric and neurodegenerative disorders [47, 48].

## 3 LC‐NE in ND and Psychiatric Disorders

**FIGURE 3:**
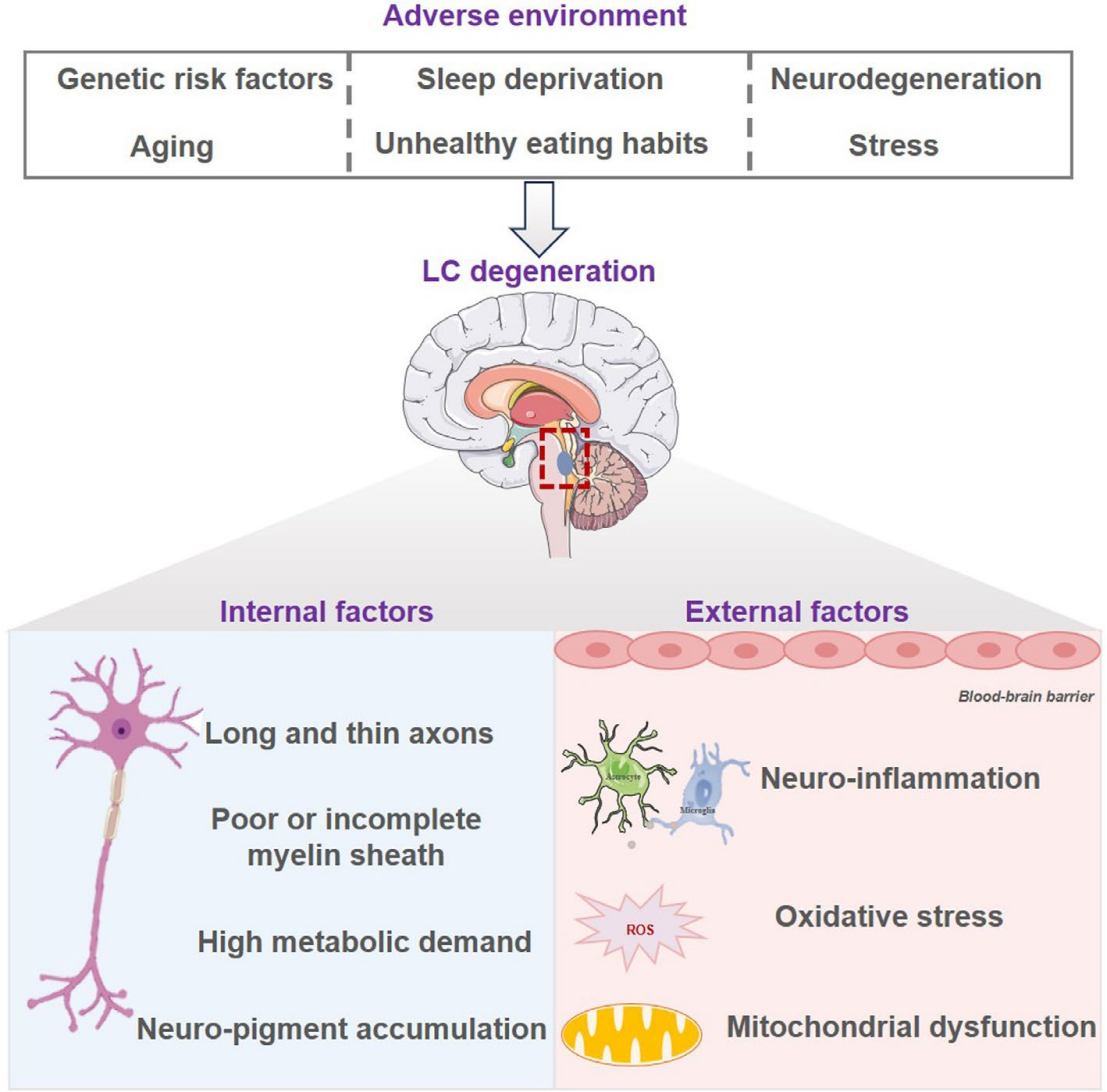
Schematic models for the selective vulnerability of LC in ND. Approximate mechanisms of vulnerability leading to LC degradation that have been proposed include internal factors, external factors and adverse environment. The extended, slender axon structure and the incomplete or inadequate myelin sheath of LC result in their heightened metabolic needs. Consequently, they are more vulnerable to the detrimental effects of internal factors like oxidative stress, mitochondrial dysfunction and neuroinflammation. In the LC, the insolubility of neuropigments impedes material transport and information transmission, subsequently resulting in a decline in cellular metabolic activity, which may further contribute to neuronal dysfunction and degeneration. Adverse environmental factors can further amplify the vulnerability to LC, including genetic predispositions, aging processes, sleep deprivation and neurodegenerative conditions.

ND is characterised by the significant degeneration of brain cells and circuits. Recent studies have highlighted early pathological changes in the LC‐NE system, indicating a strong link between LC‐NE dysfunction and ND development. One key reason for the close association is the LC's susceptibility to neuropathological changes, due to its unique cellular structures, including long, thin axons and incomplete or poorly myelinated sheaths. These structural vulnerabilities increase the energy demands of LC neurons, making them more prone to oxidative stress and neuroinflammation. Moreover, the LC innervates the vast majority of the brain's microvasculature, and any impairment of the BBB makes it more susceptible to environmental toxins. The LC's proximity to the fourth ventricle also increases the risk of exposure to cerebrospinal fluid toxins [49]. Additional factors, such as genetic predispositions, aging, sleep deprivation and neurodegeneration, further exacerbate LC vulnerability [50]. Neuromelanin‐containing catecholaminergic neurons in the LC are especially prone to developing PD‐like lesions [51]. The accumulation of insoluble neuromelanin can impair material transport, mitochondrial respiration and cellular metabolism, ultimately leading to neuronal dysfunction and degeneration [52] (Figure 3). Individuals eventually diagnosed with ND often exhibit mental symptoms, including anxiety and major depression. These psychiatric disorders are characterised by varying degrees of impairment in cognitive, emotional and behavioural mental activities, including sensory disturbances, perceptual deficits, and memory impairments [53]. Research has reported the occurrence and progression of LC‐NE disorders alongside psychiatric disorders [54]. For instance, the rostral LC is associated with memory and emotion regulation, while the caudal LC is associated with more general cognitive functions, especially in PD patients [55].

In this section, we delve into the evidence and role of LC‐NE system dysregulation in the neuropathology of ND and psychiatric disorders over the past few years. Subsequently, we summarise the similarities and differences in the roles of the LC‐NE system across different ND and psychiatric disorders. Additionally, we explore therapeutic approaches based on targeting the LC‐NE system to slow ND progression and alleviate associated psychiatric symptoms, using data from both human and animal studies (Table 1).

**TABLE 1:** Overview of evidence for pathological processes of ND and psychiatric disorders caused by LC‐NE dysfunction.

Disease	LC‐NE dysfunction	Time of dysfunction	Sample	Pathological evidence	Reference
AD	LC‐NE death	The early stages of amyloidosis pathology	Rat, AD patient	Worsening neuroinflammation, cognitive impairment, cholinergic deficiency, and neurotrophic dysregulation	[56, 57, 58]
	LC vulnerability	The advanced stages of amyloid pathology	AD patient	Accelerating the progression of AD‐like pathology, neuroinflammation, depression, anxiety, apathy, and movement disorders	[49, 58, 59]
	LC‐NE degeneration	The early stages of AD		Causing Tau protein burden, memory loss	[60, 61]
PD	LC‐NE degeneration	The early stages of PD	Mouse	Worsening learning and memory dysfunction	[62, 63]
	LC‐NE loss	Before the onset of chronic neuroinflammation		Exacerbating the gradual loss of DA and the non‐motor and motor behavioural phenotypes	[64]
	LC‐NE loss	—	PD patient	Affecting patient's attention, vigilance, and wakefulness cycles	[65, 66]
	LC‐NE degeneration	The preclinical or prodromal stages of PD		Worsening the severity of motor symptoms of PD	[66, 67]
MS	LC‐NE damage	—	Mouse	Aggravating the pathological process	[68]
	LC‐NE degeneration	—	MS patient, Mouse	Aggravating axon damage and astrocyte differentiation	[69]
MDD and Bipolar disorder	LC abnormality	—	Postmortem	Cognitive and mood disorders	[70]
RTT	LC‐NE deficiency	—	Mouse RTT patient	Aggravating RTT‐like symptoms	[71]

### 3.1 LC‐NE Dysfunction in ND

ND involves the progressive loss of vulnerable neuronal populations and myelin, leading to cognitive and behavioural dysfunction that can affect individuals across all age groups. This spans conditions from congenital leukodystrophy, which affects white matter in children, to age‐related diseases such as AD, PD and MS [72].

#### 3.1.1 LC‐NE and AD

Early studies suggest that the LC is one of the first brain regions to exhibit amyloid‐beta (Aβ) deposition in AD [73, 74]. LC neuronal shrinkage occurs well before substantial neuronal loss, with LC‐NE degeneration being evident in the early stages of AD [75, 76]. Recent imaging techniques that allow visualisation of LC in vivo using neuromelanin‐sensitive magnetic resonance imaging (MRI) have found that LC is strongly associated with aging and AD progression, indicating that changes in LC serve as an alternative and early biomarker for AD [77, 78, 79]. In the early stages of amyloidosis, LC degeneration exacerbates neuroinflammatory processes, cognitive impairment, cholinergic deficits and dysregulation of neurotrophic factors [56]. At the advanced stages of amyloid pathology, LC pathology accelerates AD progression by disrupting the neuroprotective and anti‐inflammatory properties of NE [59, 80]. In mouse models with increased Aβ production (APP‐PSEN1), Amyloid β‐oligomers (AβO) dysregulates LC activity and contribute to the spectrum of pathology of the LC‐NE system [81]. The selective vulnerability of LC neurons in AD may be attributed to NE metabolism, Tau protein cleavage and neuroinflammation [82]. NE also influences AD pathology through its effects on microglia and astrocyte function [83]. Additionally, mitochondrial dysfunction and the loss of key enzymes like glutamate pyruvate transaminase 2 (GPT2) have been implicated in early LC neurodegeneration [84]. Recent studies have linked LC degeneration to tau pathology and memory loss in AD patients [60]. Apolipoprotein E‐4 (ApoE4) exacerbates Tau pathology in AD by inhibiting vesicular monoamine transporter 2 (VMAT2) in the LC [85]. Furthermore, LC‐NE depletion in rodent AD models affects long‐term potentiation (LTP) and synaptic plasticity in the hippocampus and neocortex [86], suggesting that LC‐NE dysfunction may also disrupt other brain regions and further worsen AD progression.

#### 3.1.2 LC‐NE and PD

In PD, LC‐NE degeneration and NE signalling dysfunction are hallmark features, with post‐mortem analyses confirming LC‐NE damage [87, 88]. The LC‐NE, one of the first brain structures to be affected in PD, plays a crucial role in non‐motor symptoms. Its deficiency in disease conditions is also thought to lead to disease progression [89]. LC‐NE could stimulate neuroprotective mechanisms and regulate immune cells, while NE disorders may exacerbate disease progression, leading to chronic neuroinflammation connected with PD pathology [90]. The α‐synuclein pathology in LC is prior to substantia nigra (SN) DA in PD patients, and the early loss of LC‐NE in PD may accelerate the gradual loss of dopaminergic neurons [91, 92, 93]. Recent studies suggest that the onset of symptoms in PD patients with reduced DA‐producing A9 substantia nigra neurons may be related to the substantive pathology of norepinephrine A6 LC neurons [94]. In PD, diffuse MRI detected changes in LC and its fasciculus to the dorsolateral prefrontal cortex (DLPFC) and motor cortex (M1) that were related to local NE loss within LC, rather than cortical NE changes [79]. As a critical node in the stress response, conditional knockout of prostaglandin E2 (PGE2) receptors in LC‐NE leads to enhanced depression‐like behaviour, prolonged wakeup time in dark periods, and increased [Ca^2+^] after stress exposure, further aggravating the non‐motor symptoms of PD [95, 96]. Importantly, NE released by the LC also shapes a large and reproducible set of genes in the dorsal and ventral hippocampus during stress exposure [97]. In the paraquat and maneb‐induced mouse PD model, LC/NE neurodegeneration plays a key role in mediating learning and memory dysfunction through ferroptosis and microglia‐mediated neuroinflammation [62]. Selective deletion of LC‐NE prior to induction of chronic neuroinflammation exacerbates the non‐motor and motor behavioural phenotypes that reproduce PD symptoms [64].

#### 3.1.3 LC‐NE and MS

MS is characterised as a chronic autoimmune disorder that targets the nervous system, leading to a progressive and irreversible process of demyelination with profound motor and non‐motor implications [98, 99]. Studies have indicated that perturbations in the physiological homeostasis or signalling of the LC‐NE system may be implicated in the pathogenesis of MS. Post‐mortem examinations of human brains have revealed diminished levels of LC‐NE and marked activation of astrocytes within and surrounding the LC [69]. Furthermore, functional impairments of the LC‐NE system and axonal damage have been documented in patients diagnosed with MS [100, 101]. In line with these findings, neuronal injury in the LC and decreased levels of NE in the cortex and spinal cord have been observed in Experimental Autoimmune Encephalomyelitis (EAE) mouse models, which serve as experimental analogs for MS [68]. These collective findings suggest that alterations in the LC‐NE system may play a contributory role in the pathophysiology of MS, and therapeutic strategies targeting NE could potentially mitigate the underlying pathological processes and progression of the disease.

### 3.2 LC‐NE and Psychiatric Disorders

Research indicates a strong link between the disturbances in the NE system and various psychiatric disorders such as anxiety, depression, sleep disorders and Rett syndrome (RTT) [102, 103]. The LC‐NE system is one of the initial responders to stress, and its activation is known to induce hypervigilance and anxiety‐like behaviour [104]. Conversely, selective inhibition of LC‐NE prevents the onset of anxiety‐like behaviour following stress [105]. This is further corroborated by studies showing that mice lacking NE are immune to the anxiety‐reducing effects of sudden stress and direct stimulation of the LC via optogenetic [106]. The LC‐NE system's role in these responses may stem from the fact that activating LC‐NE fibres with light triggers the release of NE in the basolateral amygdala (BLA), which then modifies neuronal activity in the BLA, leading to negative emotions and an increase in anxiety‐related behaviours [107]. However, as LC is a heterogeneous structure, recent studies have shown that not all LC‐NE activation promotes anxiety‐like behaviour. In fact, LC neurons that connect to the medial prefrontal cortex (mPFC) appear to have the opposite effect on anxiety‐related behaviours [96]. These findings could pave the way for new approaches to treating anxiety by targeting the LC‐NE system. Major depressive disorder (MDD) is a common and debilitating mental health disorder that causes a very high risk of suicide [108]. LC dysfunction is involved in the pathophysiology of depression and is one of the most intensively studied brain regions in depression [70]. LC activation contributes to the activation of the hypothalamic–pituitary–adrenal (HPA) axis, which plays a central role in the aetiology of stress‐induced MDD [109]. The effectiveness of NE inhibitors in alleviating symptoms in depressed patients further underscores the LC's role in depression [110]. Recent studies have shown that the deletion of glucocorticoid receptors (GR) in LC‐NE prevents depression‐like behaviour in female mice, which may reveal new drug targets for the treatment of depression [111]. RTT, a disorder on the autism spectrum, arises from mutations in the X‐linked gene MeCP2, which has wide‐ranging effects. In RTT and MECP2‐deficient mice, the LC‐NE system is defective [71]. Observations in a mouse model of RTT include altered intrinsic properties of LC neurons and deficits in NE biosynthesis [112]. Understanding this link will be crucial for the development of novel medications aimed at treating psychiatric and neurological disorders.

### 3.3 LC‐NE Differences in ND and Psychiatric Disorders

The preceding sections highlight the critical role of dysregulation within the LC‐NE system in the neuropathological mechanisms underlying ND and psychiatric disorders. It is important to note that the role of LC‐NE varies across different ND and psychiatric conditions, with differences observed in the timing, extent and specific regions affected by LC dysfunction. Among ND, AD exhibits the most pronounced loss of LC‐NE, with a strong correlation between disease progression and the extent of LC‐NE loss [113]. Postmortem analysis of AD brain tissue has quantified the magnitude of LC degeneration indicate that cell loss reaches as high as 50% in the rostral region of the nucleus. Furthermore, this is correlated with a 31% reduction of cortical NE levels [114]. These is a positive correlation between the degree of LC loss and AD duration. Pathological alterations in the LC can disrupt the electrophysiological state of substantia nigra DA neurons, impair neurotransmitter metabolism and increase neuronal vulnerability, consequently contributing to the pathological progression of PD. The LC pathology precedes substantia nigra in PD and is more extensive [115]. However, unlike AD, there is no correlation between the degree of LC loss and PD duration. In contrast, MS and psychiatric disorders (such as RTT), do not show a significant loss of LC neurons comparable to that seen in AD or PD [103]. In PD, neuronal loss is relatively uniform across the rostral‐caudal extent of the nucleus, with the rostral portion being most affected, while in AD, caudal LC neurons are relatively spared [115]. A comprehensive understanding of the distinct roles of LC across various ND and psychiatric disorders may facilitate more effective targeting LC to slow ND progression and alleviate associated psychiatric symptoms.

### 3.4 Therapeutic Strategies of LC‐NE Pathways

LC‐NE dysregulation is implicated in cognitive dysfunction and the progression of ND, including PD, AD and MS. Consequently, pharmacological agents that augment NE signalling may offer therapeutic benefits in slowing the progression of ND and associated psychiatric conditions. A comprehensive review of the literature reveals important insights into the therapeutic effects of targeting the LC‐NE system in both ND and psychiatric disorders (Table 2).

**TABLE 2:** Overview of the therapeutic effects of targeted LC‐NE in ND and psychiatric disorders.

Disease	Sample	Mechanism of pathogenesis	Drug agent/therapy strategy	Function	Reference
AD	Mouse	Increasing NE levels or activity	Reboxetine	Mitigating neuroinflammation, neurodegeneration, and cerebral alterations	[116]
	AD patient		Atomoxetine	Reducing Tau and phosphorylated Tau, and alleviating mild behavioural disorders	[117]
	Mouse		Monoamine oxidase inhibitors(selegiline or rasagiline)	Improving the clinical symptoms	[118]
	Mouse	Minimising LC damage	Vindeburnol	Reducing Aβ plaque accumulation and behavioural abnormalities	[119, 120]
PD	Mouse	Increasing NE levels	Reboxetine	Suppressing neurodegeneration	[121]
MS	Mouse	Increasing NE levels	Atomoxetine and L‐DOPS	Improving the clinical symptoms of EAE mice	[68]
	MS patient		Lofepramine or Maprotiline	Relieving clinical symptoms of MS patient	[122]
	Mouse	Enhancing LC activity	Chemo‐genetic activation	Alleviating symptomatology in EAE mice	[123]
Psychiatric disorders	Rat	Inhibiting the overactivation of LC‐NE	Cannabigerol	Suppressing anxiety	[124]
	Mouse		Corticosteroid receptor blockers	Alleviating major depression	[125]
	Rat	Enhancing LC activity	Optogenetic stimulation	Enhancing memory	[126]

LC‐NE represents a promising therapeutic target for ameliorating DA deficits in the prefrontal cortex linked to neuropsychiatric pathologies [127]. Enhanced NE activity has been shown to protect against the loss of DA and maintain neuronal integrity in mice models expressing the human α‐synuclein A53T mutation, thereby preventing motor dysfunction [128]. The overexpression of transcription factors Phox2a/2b, Hand2 and Gata3 in LC improves NE and DA activity and function in PD rodent models, offering a therapeutic strategy for PD treatment [92].

Pharmaceutical interventions targeting NE have also been observed to influence the course of ND. For instance, reboxetine, a NE reuptake inhibitor, may improve the PD phenotype related to delayed progression of substantia nigra (SN)/ventral tegmental area (VTA) dopaminergic neurodegeneration as well as higher DA levels in the striatum [121]. Additionally, reboxetine mitigates neuroinflammation, neuro‐degeneration and counteracts the cerebral alterations induced by excessive amyloid β production in 5 × FAD mice [116]. Atomoxetine, an approved NE transporter inhibitor, not only reduces Tau and phosphorylated Tau in cerebrospinal fluid, but also normalises a group of protein biomarkers associated with synaptic function, brain metabolism and immunity, alleviating mild behavioural disorders [117]. The synergistic use of atomoxetine with the synthetic norepinephrine precursor L‐threo‐3,4‐dihydroxyphenylserine (L‐DOPS) effectively improves the clinical symptoms of EAE mice [68]. Moreover, the combination of NE reuptake inhibitors (lofepramine or maprotiline) with levodopa presents therapeutic advantages for MS patients [122]. Monoamine oxidase inhibitors (MAOI) like selegiline or rasagiline further contribute to AD and PD management by elevating brain NE levels [118, 129].

Emerging research on cannabigerol (CBG) reveals its potential to block the inhibitory action of selective α2‐adrenergic receptors on LC‐NE firing rates, producing anxiolytic effects via the 5‐HT1A receptor in rats [124]. The loss of ErbB4 (a receptor tyrosine kinase) in LC‐NE leads to hyperactivity and anxiety, indicating its therapeutic relevance [130]. Beyond anxiety, targeting LC‐NE is also beneficial for depression. For example, fast‐acting antidepressants inhibit the over‐activation of LC‐NE and alleviate the disease process [109]. Both Phox2a and Phox2b proteins are highly expressed in LC in the brains of patients with major depression. Corticosteroid receptor blockers inhibit the expression of Phox2a/b and then regulate the norepinephrine phenotype to alleviate major depression [125]. Ginsenoside Rg1 has been found to promote sleep in rats by regulating the LC‐NE and the serotonergic system of the dorsal septal nucleus [131].

Rather than solely increasing NE levels or activity, strategies that minimise LC neuronal damage or enhance LC neuronal activity may be more advantageous in curtailing ND progression. Vindeburnol, for example, reduces Aβ plaque accumulation and behavioural abnormalities by restoring the expression of TH that is lost during development in LC neurons [119, 120]. Activation of TH neurons in LC alleviates depression‐like behaviour in susceptible mice, possibly through brain‐derived neurotrophic factor (BDNF) [132]. Vagus nerve stimulation, which enhances LC neuronal activity, increases NE levels and BDNF expression in the brain [133]. Optogenetic stimulation of the LC in rats enhances memory and determines the likelihood of sensory‐induced arousal [14, 126]. Selective stimulation of NE neurons in the LC in transgenic mice by a chemo‐genetic activation system enhances the retrieval of conditioned taste aversion and changes the sleep microarchitecture similar to stress [134, 135]. Chemo‐genetic activation of LC‐NE produces anti‐inflammatory and neuroprotective effects as well as alleviates symptomatology in EAE mice [123].

In summary, these studies underscore the significant therapeutic potential of LC‐NE‐based interventions in halting ND progression. However, the early and extensive degeneration of the LC‐NE system in many ND cases presents significant challenges for direct targeting. Thus, restoring LC‐NE function via regeneration using PSCs‐derived or transdifferentiated LC‐NE neurons may offer a promising solution for future therapies.

## 4 Regeneration of LC‐NE in ND

ND, such as PD, AD and MS, result from progressive neuronal loss in the brain. Regrettably, existing treatments fail to halt the neurodegenerative processes effectively. Stem cell therapy, also referred to as regenerative cell therapy, has emerged as a powerful strategy for addressing these challenging conditions. Stem cells possess the remarkable ability to repair injured neuronal tissue by replacing damaged or lost cells, creating a conducive environment for regeneration, or protecting existing healthy neurons and glial cells from further harm.

LC‐NE dysfunction is closely associated with various ND, although the precise role of LC‐NE in these disorders remains poorly understood due to the lack of suitable cellular models that mimic human LC‐NE. The emergence of stem cell therapy offers a promising avenue for ND treatment. If LC‐NE regeneration can be achieved through stem cell differentiation, it could revolutionise therapeutic strategies. In this section, we provide a succinct introduction to the basic background of stem cells and review recent literature on stem cell therapy for ND, including AD, PD and MS. We also explore the therapeutic potential of PSC‐derived LC‐NE neurons in treating ND.

### 4.1 PSCs and the Progress of Cell Therapies in Treating ND

PSCs hold immense potential in the realm of regenerative medicine due to their capacity for indefinite proliferation and differentiation into diverse cell types. Human embryonic stem cells (ESCs) were first isolated in 1998 [136]. Despite the ethical considerations associated with the utilisation of human ESCs (hESCs), they remain one of the most reliable stem cell sources for transplantation therapies and have shown promise in treating neurological disorders. Induced pluripotent stem cells (iPSCs), reprogrammed from somatic cells via the overexpression of specific transcription factors [137], offer a versatile alternative that circumvents both immune rejection and ethical dilemmas. ESC and iPSC are widely used in clinical treatment, including organ regeneration, cell and tissue transplantation, tissue repair and drug testing [138]. PSCs are also used to model diseases and screen drugs based on three‐dimensional (3D) culture techniques that allow multiple cell types to be studied in an internal environment similar to human pathological and physiological conditions [139]. Given the complex pathogenesis and the inability of small animal models to replicate the unique characteristics of the human nervous system, ND currently lacks effective therapeutic targets in the clinic [140]. PSCs are revolutionising the landscape of ND treatment. By generating neuronal cells from individual patients, stem cell‐based therapies offer a promising solution to the species‐specific limitations of animal models and pave the way for the development of effective treatments for various NDs (Table 3).

**TABLE 3:** Advances in cell transplantation in ND.

Disease	Sample	Donor cells	Therapeutic effect	Reference
AD	Mouse	MSC	Alleviating the pathological features of AD in mice by reducing mitochondrial oxidative stress	[141]
		iPSC	Reducing Aβ plaque deposition and improving cognitive impairment	[142]
		iNPC	Enhancing synaptic plasticity and cognitive abilities	[143]
	AD patient	MSC	Improving the clinical symptoms	[144, 145]
PD	Mouse	hPSC	Improving the motor symptoms	[146]
	PD patient	NSC	Alleviating the clinical symptoms	[147, 148]
MS	Mouse	MSC	Improving clinical scores and relieving inflammation	[149]
	MS patient	NPC	Promoting neuroprotection and remyelination	[150]
		AHSC	Delaying disability progression in patients with active secondary progressive multiple sclerosis (SPMS)	[151]

#### 4.1.1 Cell Therapies for AD

AD is marked by extracellular deposition of Aβ plaques and intracellular neurofibrillary tangles (NFT) of neurons with hyperphosphorylated tau protein. Despite the lack of effective disease‐modifying therapies in clinical practise, stem cell transplantation offers new perspectives on AD pathogenesis and potential treatments. Transplantation of mesenchymal stem cells (MSC) or MSC‐conditioned medium (MSC‐CM) alleviates the pathological features of AD in mice by reducing mitochondrial oxidative stress [141, 152]. MSC‐extracellular vesicles (EVs), as important intercellular communication mediators, have great potential in the treatment of AD, including the elimination of abnormal protein accumulation, neuroprotective and immunomodulatory effects [153]. Studies have shown that intranasal delivery of EVs derived from MSC plays immunomodulatory and neuroprotective roles in mouse models of AD [154]. Completed or ongoing clinical trials using various sources of MSCs have validated their safety and efficacy [144]. Studies have shown that protein‐iPSCs, which are derived from mouse skin fibroblasts by treating protein extracts of ESCs, are pluripotent but nontumorigenic. Transplantation of protein‐iPSCs reduces Aβ plaque deposition and improves cognitive impairment in 5 × FAD mice [142]. Although these studies consistently report improved cognitive performance in AD animal models following stem cell transplantation, few have investigated synaptic and neural circuit changes post‐transplantation. Recent research by Jing et al. sheds light on this question: functional human‐induced neural progenitor/stem cells (iNPCs) transplanted into the hippocampus of immunodeficient AD mice enhance synaptic plasticity and cognitive abilities [143]. Additionally, enhanced microglial replacement after whole‐body haematopoietic cell transplantation restores microglial function in AD mouse models with Trem2 mutations [155]. However, key questions remain, including how stem cells can rescue cognitive deficits and their suitability for replacement therapy. Addressing challenges such as safety, functional integration of transplanted cells, optimal animal models and immune rejection will be crucial in advancing this therapeutic approach.

#### 4.1.2 Cell Therapies for PD

In PD, the A9 DA in the substantia nigra of the ventral midbrain (VM) project to the striatum degenerate, resulting in movement disorders. VM cells derived from hPSC produce DA rich in A9. When transplanted into animals, they stably exist and function, and further improve the motor symptoms of PD mice [146]. Currently, clinical trials are investigating cell replacement therapy for PD using stem cell derived DA neurons. The first autologous transplantation in patients with PD has been carried out [147]. Recently, a phase 1 clinical study has achieved a breakthrough in demonstrating the safety and preliminary efficacy of human NSCs in treating PD. This study marks the first global instance of transplanting NSCs through the nasal mucosa to treat PD, a method shown to be feasible, generally safe and well tolerated [148]. Additionally, preclinical studies have confirmed the quality, safety and efficacy of PSC‐derived products for PD treatment [156, 157, 158, 159]. However, the surgical procedure may induce a profound host response, including acute neuroinflammation, intense infiltration of peripheral immune cells and brain cell death, resulting in a low survival rate of transplanted DA neurons. Recent studies suggest that co‐transplantation of Treg cells and hiPSC‐derived midbrain dopamine (mDA) cells into the striatum significantly improves the therapeutic effects of PD rodent models by preventing damage to the transplanted mDA neurons [160]. Moreover, a pooled CRISPR‐Cas9 screen identified p53‐mediated apoptosis as a key contributor to DA neuron loss, highlighting TNF‐NF‐κB signalling as a limiting factor for cell survival. Transient inhibition of TNF‐α improved the survival of postmitotic hPSC‐derived DA in PD models [161]. While autologous transplantation has shown promise in avoiding immunosuppression, challenges such as heterogeneity in hPSC‐derived cells and low production of mDA neurons following transplantation need to be addressed to ensure clinical success. Ongoing research is focused on enhancing the differentiation and survival of mDA neurons post‐transplantation, with autologous transplantation in PD patients showing promising results without the need for immunosuppression [162, 163]. Specific surface markers of mDA progenitors, such as Calsyntenin 2 (CLSTN2) and protein tyrosine phosphatase receptor type O (PTPRO), improve progenitor cell sorting and mDA neuron enrichment after transplantation, enhancing the prospects of hPSC‐based PD cell replacement therapy [164]. Another innovative strategy involves the direct reprogramming of fibroblasts and astrocytes into neurons [165]. Advances in stem cell biology have enabled CRISPR/Cas9‐mediated editing of ESCs and patient‐derived stem cells in vitro, leading to personalised cell therapies for PD [166]. In summary, direct differentiation of PSC or trans differentiation of glia cells into DA neurons have shown promising results in the treatment of PD, but further efforts are needed to enhance their efficiency and improve their survival post‐transplantation or conversion to achieve stable and predictable therapeutic outcomes.

#### 4.1.3 Cell Therapies for MS

MS has been recognised as an autoimmune disease with dual characteristics of neuroinflammatory and neurodegenerative lesions. While existing treatments can slow the course of the disease, a cure remains elusive [167]. Stem cell transplantation not only provides neurotrophic support, immune modulation and cell replacement, but also shows great promise in combating the complex pathology of chronic neuroinflammation, thus undoubtedly becoming a therapeutic strategy for MS. Transplanting MSC into the peritoneum of EAE mice effectively improves clinical scores and relieve inflammation [149]. Neural precursor cells (NPCs) have shown preclinical efficacy when transplanted into animal models of MS to promote neuroprotection and remyelination by releasing molecules that maintain nutritional support and neuroplasticity, and this treatment regimen is feasible, safe and tolerable [150]. Recent results have also confirmed that autologous haematopoietic stem cell transplantation (AHSCT) delays disability progression in patients with active secondary progressive multiple sclerosis (SPMS). Importantly, AHSCT is more likely to improve disability than standard immunotherapy [151, 168]. Moreover, the development of hypoimmunogenic human iPSC‐derived oligodendroglial progenitor cells (OPCs) represents a promising cell therapy for myelin regeneration, offering potential treatments for myelination disorders and motor deficits [169]. The differentiation of human neural stem cells (HNSCs) into human OPCs and their subsequent transplantation into the entire major white matter tract is also found to effectively promote myelin regeneration of axons in the corpus callosum [170]. When Olig2‐induced OPC is transplanted into transient middle cerebral artery occlusion (tMCAO) rat models, it reduces neuronal death, promotes myelin regeneration and salvage spatial memory decline as well as effectively promotes neural function recovery [171]. Other relevant studies have been reviewed elsewhere [172, 173, 174].

### 4.2 Generation of LC Neurons From PSCs

Given the facts that LC‐NE disorders are inextricably related to ND, and targeting to improve LC‐NE activity or reduce LC damage has beneficial effects in ND treatment. With the emergence of stem cell therapy in ND treatment, we have reason to believe that if the regeneration of LC‐NE is achieved through PSCs, it will provide a promising therapeutic strategy for ND treatment.

#### 4.2.1 LC Development in Animal Models

To achieve LC‐NE regeneration, a thorough analysis of its origin and development is essential. Initial cross‐fate mapping experiments demonstrate that LC is mainly derived from Engrailed‐1 (En1) expression domains containing hindbrain rhombomere 1(R1), with a small subpopulation derived from R2 [36, 175]. Early animal studies indicated that LC‐NE neurons originate predominantly from the hindbrain R1 region, whereas other NE nuclei develop from R2–R5 [175]. However, it remains plausible that they initially arise in one domain and subsequently migrate between neurons from another. Recent single‐gene fate mapping using fibroblast growth factor 8 (Fgf8)‐Cre reveals that NE from r0 is involved in dorsal LC, and NE from r1 is involved in ventral LC [176]. Recent findings have utilised spatially resolved transcriptomics and single nuclear RNA sequencing to characterise the molecular landscape of the human brain's LC region and the transcriptomic features of LC‐NE, providing further insights into the development of LC [21]. However, the study of LC‐NE systems is still hampered by the lack of available authentic NE neurons, particularly those of human origin.

#### 4.2.2 Generation of LC‐NE Neurons From hPSCs

Existing “NE‐like” cell lines, such as PC12 and SH‐SY5Y, express certain gene profiles and produce NE neurotransmitters. However, they fall short of replicating authentic developmental processes and often harbour mutations that diverge from true NE neuron characteristics [177]. Specific types of neurons can be generated by the forced expression of certain transcription factors in a neural progenitor cell derived from ESC [178]. Using this strategy, the researchers induce mouse ESCs (mESCs) and hESC to generate norepinephrine neurons through forced expression of homeobox transcription factor Phox2b under the signalling influence of FGF8 and bone morphogenetic protein [179]. Unfortunately, the expression of TH and DA‐β hydroxylase (DBH) produced in hPSC, markers of NE neurons, is much lower than that observed in mESC. Notably, species differences in stem cell differentiation pathways to NE have been observed. For instance, bone morphogenic protein 7 (BMP7) facilitates NE neuronal generation in mice but impedes it in human cells [179, 180]. Consequently, the differentiation pathway of LC‐NE neurons remains elusive, necessitating novel methodologies to induce hPSCs to differentiate into central NE‐potent neurons.

Based on the development of LC‐NE learned from animal studies, Tao et al. first differentiated hPSCs into neuroepithelial cells in the R1 region, but the R1 neuroepithelial cells contained very few LC‐NE progenitor cells expressing ASCL1, PHOX2B. Through extensive screening, the researchers discovered that Activin A effectively induces the specification of LC‐NE progenitors. These NE neurons, generated from LC‐NE progenitors, exhibit in vivo‐like characteristics, including the synthesis, release and reuptake of norepinephrine, extensive axonal branching and CO_2_ sensing, confirming their functionality.

To validate these PSC‐derived NE neurons, the researchers conducted a multi‐time point single‐cell sequencing experiment to dynamically analyse the composition of the differentiated cells and their differentiation pathway and confirmed the expression of molecular markers of NE and their proportions. Moreover, when compared with single‐cell data from in vivo tissues, the gene expression profile of these NE neurons closely resembled that of cells in the LC region of embryonic mice, further validating the cell type. This use of PSCs to differentiate NE neurons in the LC holds immense promise for advancing basic research in psychiatric and ND and offers new avenues for therapeutic development [181].

### 4.3 Exploring the Cell Therapy of ND by Targeting LC Regeneration via Stem Cells

**FIGURE 4:**
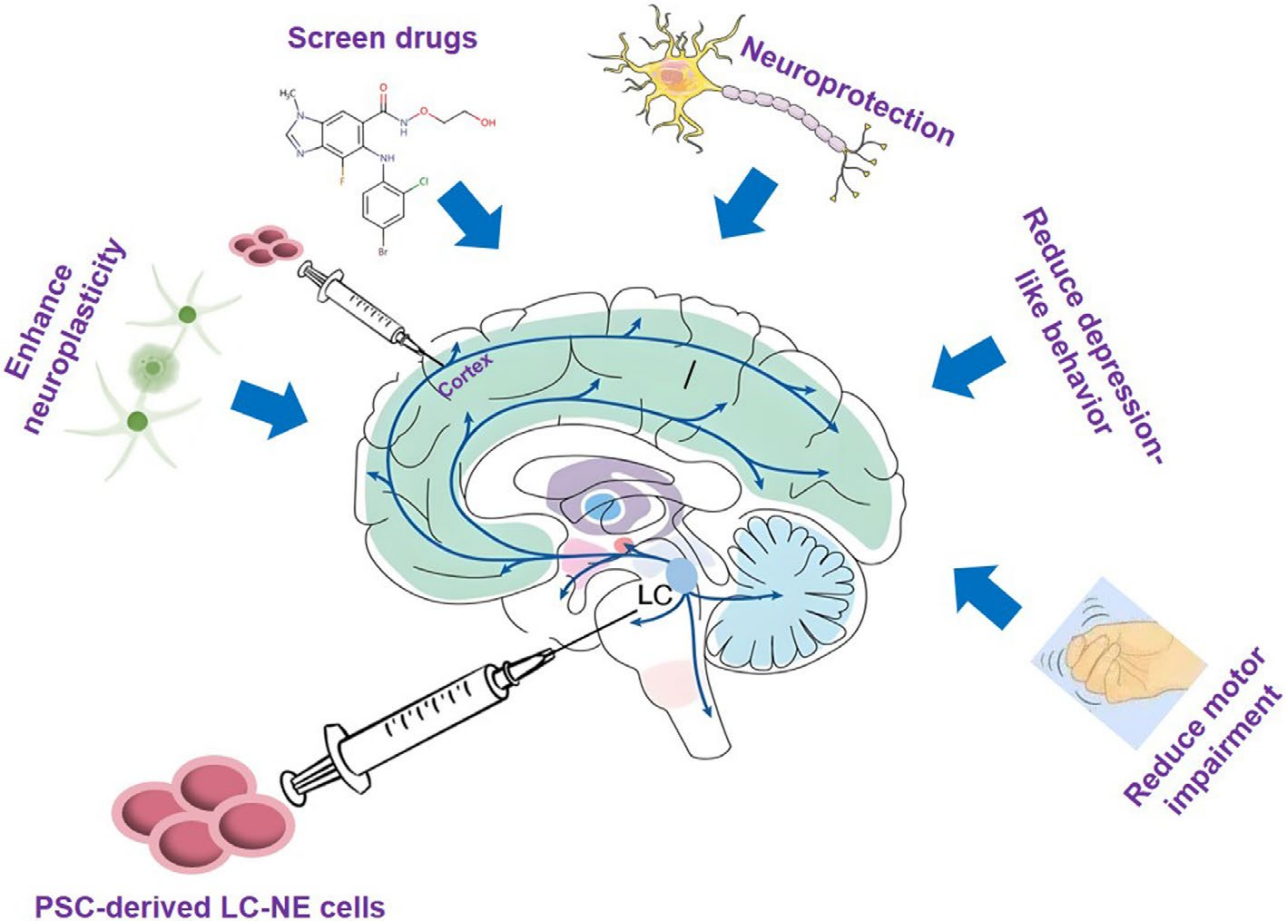
Schematic diagram of the function of LC‐NE transplantation. If stem cell‐derived LC‐NE can be successfully transplanted, whether through orthotopic transplantation or in alternative sites (e.g., the cortex), it has the potential to alleviate associated ND and psychiatric symptoms, including alleviating motor impairments and diminishing depression‐like behaviours, while simultaneously providing neuroprotective effects and enhancing synaptic plasticity. The hPSC‐derived LC‐NE cell platform can be used to screen drugs to treat neurological disorders.

The LC‐NE system's involvement in the pathogenesis of various ND positions it as a prime candidate for novel pharmacological interventions. Historically, the absence of adequate in vitro LC‐NE models and a limited understanding of LC‐NE vulnerability in disease mechanisms have impeded research progress. The advent of hPSC‐derived LC‐NE neurons heralds a new era, offering potential solutions to these challenges and paving the way for LC‐targeted cell therapies for ND (Figure 4).

The hPSC‐derived LC‐NE cell platform can be used to screen drugs to treat neurological disorders, resulting from disruption of the norepinephrine neurotransmitter, such as depression. The study found that drugs targeting the recovery process of norepinephrine neurotransmitter significantly improve the signal intensity of NE sensor, and can be used as candidate drugs for depression [181]. Beyond drug discovery, hPSC‐derived LC‐NE are anticipated to serve as direct therapeutic agents for ND. DSP4, a selective neurotoxin for the LC, enables the rapid and non‐invasive simulation of LC loss [182], providing a valuable model for evaluating the therapeutic potential of hPSC‐derived LC‐NE in ND.

Successful cell therapy hinges on the transplanted cells' ability to replace lost neurons and reestablish functional neural circuits, making the transplantation site a critical factor. The importance of the graft site is further illustrated by examples where anatomical presynaptic inputs largely depend on whether hESC‐derived midbrain DA or cortical glutamate neurons are transplanted into the substantia nigra or the striatum of a mouse model of PD [183]. Gene‐labelling strategies can be employed to map the projections and synaptic inputs of LC‐NE neurons accurately.

Ultimately, the therapeutic efficacy of LC‐NE neurons following transplantation will depend on their ability to restore phenotype and function, including the expression of key proteins, neuronal electrophysiological characteristics, and the recovery of motor and cognitive function. While the development of LC‐targeted cell therapies for ND holds great promise, significant research and development efforts are still needed to optimise these strategies for future clinical applications.

## 5 Future Direction

The feasibility of LC‐NE transplantation has been studied. Transplantation of LC‐NE from fetal rat donors into aged rats can ameliorate avoidance deficits [184]. LC/hippocampal co‐grafts generated from foetuses expressing the wildtype glial cell line‐derived neurotrophic factor (GDNF) background can survive normally after transplantation into the brains of adult wild‐type mice [185]. These results provide empirical support for the feasibility of hPSC‐derived LC‐NE cell in transplantation therapies. Despite significant advancements in LC‐NE regeneration, several pivotal challenges remain to be addressed in the future. The hindbrain houses various subtypes of NE neurons, with LC‐NE neurons originating mainly from the hindbrain's R1 region [36, 186]. One critical goal is the efficient generation of neuroepithelial cells with a posterior R1 identity while avoiding the induction of other NE subtypes originating from regions R2 to R5. This selective differentiation is crucial for enhancing the efficiency of LC‐NE neuron production. The activation of the WNT pathway by CHIR99021 exerts a dose‐dependent influence on the neuroepithelial patterning, spanning from the anterior to the posterior axis. Fine‐tuning its concentration could lead to the efficient generation of R1 progenitor cells in the posterior brain, potentially serving as an effective strategy for inducing the production of specific NE subtypes [187]. The generation of LC‐NE from hPSCs is crucial, but often not sufficient. Further contemplation should be given to evaluating the function of NE and its therapeutic impact on ND using animal models. The selection of suitable animal models, delivery methods and transplant sites is crucial for maximising therapeutic efficacy and improving outcomes. Overcoming the potentially fatal consequences of brain stem or other transplants is a necessity. It is worth noting that while animal models are designed to maximise reproducibility, there is considerable variability and diversity among human patients. Translating the results of these in vivo studies to human patients presents a formidable challenge. At the same time, as previously mentioned, LC has a unique cellular structure, including long, thin axons and incomplete or poorly myelinated sheaths, making them more vulnerable in ND pathology. Stem cell‐derived LC‐NE will may also be affected by these conditions. However, stem cell derived neurons are relatively young in development, which may make them more resisting to environmental stresses since LC degeneration in ND used to happen in aged individuals instead of young people. Additionally, to further improve their survive post‐transplantation, it may be possible to perform the co‐transplantation of LC‐NE with oligodendrocytes to form myelinated LC‐NE cells.

A variety of therapeutic approaches, including neuromodulation techniques, gene therapy, environmental enrichment and behavioural interventions, show promise in regenerating LC‐NE functions. Neuromodulation and optogenetic stimulation by LC‐NE have a powerful effect on general arousal and wakefulness [188]. For example, environmental enrichment is known to be beneficial for cognitive improvement, which increases the expression of pro‐regeneration genes and promotes axonal regeneration [189]. Injecting Designer Receptors Exclusively Activated by Designer Drugs (DREADDs) into LC by adeno‐associated viruses to selectively stimulate LC neurons by exogenous administration of the inert DREADDs ligand clozapine‐N‐oxide prevented memory loss and improved mobility in Ts65Dn mice [190]. Although these methods possess certain effects and advantages, they may have minimal efficacy in pathological situations where LC degeneration or loss has occurred.

The advent of hPSC‐derived LC‐NE cells has provided unprecedented capabilities for studying the pathogenesis of ND in vitro and to screen drugs for the treatment of neurological diseases caused by the disruption of NE metabolism, such as depression. Although cell therapy for ND offers distinct advantages, before hPSC‐derived LC‐NE is applied to treat ND, further research is needed to ensure the safety of the treatment. One of the key problems in current stem cell transplantation therapy is the immune rejection of transplanted cells. Solving the problem of immune rejection of grafted cells requires consideration of the appropriate donor cell type, dose and duration of immunosuppressive drug treatment. The other challenge regarding the application of hPSC‐derived LC‐NE replacement strategies for ND is that grafted cells may become diseased in a diseased brain microenvironment. In this case, the transplanted cells may acquire corresponding pathology over time, leading to their loss of function and eventually their death. Additionally, the differentiated cell populations acquired through current approaches might contain other cell types, giving rise to heterogeneity. Such heterogeneity could result in tumour formation in the host following transplantation, thereby presenting a potential and unpredictable safety risks for patients. Particularly, additional risks include challenges specific to brainstem transplantation, the potential for overdose, broad projections within the brain and stem cell migration to inappropriate regions of the body in the brain also need to be taken into account in the LC transplantation.

As discussed, the LC‐NE system functions differently across ND and psychiatric disorders. On one hand, LC‐NE hyperactivity can contribute to anxiety‐like behaviours in psychiatric disorders, while LC‐NE deficits can be associated with conditions such as RTT. Therefore, the application of hPSC‐derived LC‐NE cell therapy for psychiatric disorders requires careful targeting LC‐NE projections or related circuits in psychiatric or neurodegenerative conditions may be affected differentially as well, necessitating tailored transplantation sites. For example, in RTT, LC‐NE function is impaired but not completely lost, so the required transplant dosage would differ from that for ND and must be carefully calibrated.

Despite all the limitations and challenges, cell therapies that regenerate LC‐NE function remain a promising approach for treating ND and psychiatric disorders in the future. Continued research and innovation in this field are essential to overcoming existing obstacles and advancing towards effective clinical applications.

## 6 Conclusions

Stem cell technologies present a tangible avenue towards the development of effective treatments for ND. By elucidating the mechanisms that underpin the dysfunction of the LC‐NE system and capitalising on the potential of PSCs‐derived LC‐NE, we can lay the groundwork for pioneering therapies. These innovative treatments not only alleviate the symptoms but also hinder the progression of the disease, thereby offering a promising outlook for the management of ND.

## Author Contributions

Y.N.Y. drafted the manuscript, and illustrated the figures and tables. Y.L.T. acquired the funding, conceived and revised the manuscript.

## Conflicts of Interest

The authors declare no conflicts of interest.

## Data Availability

The data that support the findings of this study are available from the corresponding author upon reasonable request.

## References

[1] V. Breton‐Provencher , G. T. Drummond , and M. Sur , “Locus Coeruleus Norepinephrine in Learned Behavior: Anatomical Modularity and Spatiotemporal Integration in Targets,” Frontiers in Neural Circuits 15 (2021): 638007. 34163331 10.3389/fncir.2021.638007 PMC8215268

[2] D. L. Feinstein , S. Kalinin , and D. Braun , “Causes, Consequences, and Cures for Neuroinflammation Mediated via the Locus Coeruleus: Noradrenergic Signaling System,” Journal of Neurochemistry 139, no. Suppl 2 (2016): 154–178. 26968403 10.1111/jnc.13447

[3] F. Sivandzade and L. Cucullo , “In‐Vitro Blood‐Brain Barrier Modeling: A Review of Modern and Fast‐Advancing Technologies,” Journal of Cerebral Blood Flow and Metabolism: Official Journal of the International Society of Cerebral Blood Flow and Metabolism 38, no. 10 (2018): 1667–1681. 30058456 10.1177/0271678X18788769 PMC6168917

[4] F. Sivandzade and L. Cucullo , “Regenerative Stem Cell Therapy for Neurodegenerative Diseases: An Overview,” International Journal of Molecular Sciences 22, no. 4 (2021): 2153–2173. 33671500 10.3390/ijms22042153 PMC7926761

[5] A. Dahlström and K. Fuxe , “Localization of Monoamines in the Lower Brain Stem,” Experientia 20, no. 7 (1964): 398–399. 5856530 10.1007/BF02147990

[6] Y. Sharma , T. Xu , W. M. Graf , et al., “Comparative Anatomy of the Locus Coeruleus in Humans and Nonhuman Primates,” Journal of Comparative Neurology 518, no. 7 (2010): 963–971. 20127761 10.1002/cne.22249 PMC2820586

[7] P. R. Mouton , B. Pakkenberg , H. J. G. Gundersen , and D. L. Price , “Absolute Number and Size of Pigmented Locus Coeruleus Neurons in Young and Aged Individuals,” Journal of Chemical Neuroanatomy 7, no. 3 (1994): 185–190. 7848573 10.1016/0891-0618(94)90028-0

[8] K. Satoh , M. Tohyama , K. Yamamoto , T. Sakumoto , and N. Shimizu , “Noradrenaline Innervation of the Spinal Cord Studied by the Horseradish Peroxidase Method Combined With Monoamine Oxidase Staining,” Experimental Brain Research 30, no. 2–3 (1977): 175–186. 74341 10.1007/BF00237249

[9] S. E. Loughlin , S. L. Foote , and R. Grzanna , “Efferent Projections of Nucleus Locus Coeruleus: Morphologic Subpopulations Have Different Efferent Targets,” Neuroscience 18, no. 2 (1986): 307–319. 3736861 10.1016/0306-4522(86)90156-9

[10] V. M. Pickel , M. Segal , and F. E. Bloom , “A Radioautographic Study of the Efferent Pathways of the Nucleus Locus Coeruleus,” Journal of Comparative Neurology 155, no. 1 (1974): 15–42. 4836061 10.1002/cne.901550103

[11] S. J. Sara and S. Bouret , “Orienting and Reorienting: The Locus Coeruleus Mediates Cognition Through Arousal,” Neuron 76, no. 1 (2012): 130–141. 23040811 10.1016/j.neuron.2012.09.011

[12] S. J. Sara , “The Locus Coeruleus and Noradrenergic Modulation of Cognition,” Nature Reviews. Neuroscience 10, no. 3 (2009): 211–223. 19190638 10.1038/nrn2573

[13] Y. U. Liu , Y. Ying , Y. Li , et al., “Neuronal Network Activity Controls Microglial Process Surveillance in Awake Mice via Norepinephrine Signaling,” Nature Neuroscience 22, no. 11 (2019): 1771–1781. 31636449 10.1038/s41593-019-0511-3 PMC6858573

[14] H. Hayat , N. Regev , N. Matosevich , et al., “ *Locus Coeruleus Norepinephrine Activity Mediates Sensory‐Evoked Awakenings From Slee*Science,” Advances 6, no. 15 (2020): eaaz4232. 10.1126/sciadv.aaz4232 PMC7141817 32285002

[15] V. Breton‐Provencher , G. T. Drummond , J. Feng , Y. Li , and M. Sur , “Spatiotemporal Dynamics of Noradrenaline During Learned Behaviour,” Nature 606, no. 7915 (2022): 732–738. 35650441 10.1038/s41586-022-04782-2 PMC9837982

[16] E. E. Benarroch , “Locus coeruleus,” Cell and Tissue Research 373, no. 1 (2018): 221–232. 28687925 10.1007/s00441-017-2649-1

[17] G. R. Poe , S. Foote , O. Eschenko , et al., “Locus Coeruleus: A New Look at the Blue Spot,” Nature Reviews. Neuroscience 21, no. 11 (2020): 644–659. 32943779 10.1038/s41583-020-0360-9 PMC8991985

[18] D. J. Chandler , P. Jensen , J. G. McCall , A. E. Pickering , L. A. Schwarz , and N. K. Totah , “Redefining Noradrenergic Neuromodulation of Behavior: Impacts of a Modular Locus Coeruleus Architecture,” Journal of Neuroscience: The Official Journal of the Society for Neuroscience 39, no. 42 (2019): 8239–8249. 31619493 10.1523/JNEUROSCI.1164-19.2019 PMC6794927

[19] N. K. Totah , R. M. Neves , S. Panzeri , N. K. Logothetis , and O. Eschenko , “The Locus Coeruleus Is a Complex and Differentiated Neuromodulatory System,” Neuron 99, no. 5 (2018): 1055–1068.e6. 30122373 10.1016/j.neuron.2018.07.037

[20] B. Mulvey , D. L. Bhatti , S. Gyawali , et al., “Molecular and Functional Sex Differences of Noradrenergic Neurons in the Mouse Locus Coeruleus,” Cell Reports 23, no. 8 (2018): 2225–2235. 29791834 10.1016/j.celrep.2018.04.054 PMC6070358

[21] L. M. Weber , H. R. Divecha , M. N. Tran , et al., “The Gene Expression Landscape of the Human Locus Coeruleus Revealed by Single‐Nucleus and Spatially‐Resolved Transcriptomics,” eLife 12 (2024): 12. 10.7554/eLife.84628 PMC10945708 38266073

[22] S. C. Kelly , B. He , S. E. Perez , S. D. Ginsberg , E. J. Mufson , and S. E. Counts , “Locus Coeruleus Cellular and Molecular Pathology During the Progression of Alzheimer's Disease,” Acta Neuropathologica Communications 5, no. 1 (2017): 8. 28109312 10.1186/s40478-017-0411-2 PMC5251221

[23] L. W. Swanson , “The Locus Coeruleus: A Cytoarchitectonic, Golgi and Immunohistochemical Study in the Albino Rat,” Brain Research 110, no. 1 (1976): 39–56. 776360 10.1016/0006-8993(76)90207-9

[24] V. R. Holets , T. Hökfelt , Å. Rökaeus , L. Terenius , and M. Goldstein , “Locus Coeruleus Neurons in the Rat Containing Neuropeptide Y, Tyrosine Hydroxylase or Galanin and Their Efferent Projections to the Spinal Cord, Cerebral Cortex and Hypothalamus,” Neuroscience 24, no. 3 (1988): 893–906. 2454419 10.1016/0306-4522(88)90076-0

[25] L. A. Schwarz and L. Luo , “Organization of the Locus Coeruleus‐Norepinephrine System,” Current Biology: CB 25, no. 21 (2015): R1051–R1056. 26528750 10.1016/j.cub.2015.09.039

[26] W. S. Young and M. J. Kuhar , “Noradrenergic Alpha 1 and Alpha 2 Receptors: Light Microscopic Autoradiographic Localization,” Proceedings of the National Academy of Sciences of the United States of America 77, no. 3 (1980): 1696–1700. 6246501 10.1073/pnas.77.3.1696 PMC348564

[27] T. M. Egan and R. A. North , “Actions of Acetylcholine and Nicotine on Rat Locus Coeruleus Neurons In Vitro,” Neuroscience 19, no. 2 (1986): 565–571. 2430232 10.1016/0306-4522(86)90281-2

[28] J. M. Luque , P. Malherbe , and J. G. Richards , “Localization of GABAA Receptor Subunit mRNAs in the Rat Locus Coeruleus,” Brain Research. Molecular Brain Research 24, no. 1–4 (1994): 219–226. 7968361 10.1016/0169-328x(94)90135-x

[29] J. N. Marcus , C. J. Aschkenasi , C. E. Lee , et al., “Differential Expression of Orexin Receptors 1 and 2 in the Rat Brain,” Journal of Comparative Neurology 435, no. 1 (2001): 6–25. 11370008 10.1002/cne.1190

[30] A. Mansour , C. A. Fox , S. Burke , et al., “Mu, Delta, and Kappa Opioid Receptor mRNA Expression in the Rat CNS: An In Situ Hybridization Study,” Journal of Comparative Neurology 350, no. 3 (1994): 412–438. 7884049 10.1002/cne.903500307

[31] C. Léna , A. de Kerchove d'Exaerde , M. Cordero‐Erausquin , N. le Novère , M. del Mar Arroyo‐Jimenez , and J. P. Changeux , “Diversity and Distribution of Nicotinic Acetylcholine Receptors in the Locus Ceruleus Neurons,” Proceedings of the National Academy of Sciences of the United States of America 96, no. 21 (1999): 12126–12131. 10518587 10.1073/pnas.96.21.12126 PMC18423

[32] G. Eisenhofer , I. J. Kopin , and D. S. Goldstein , “Catecholamine Metabolism: A Contemporary View With Implications for Physiology and Medicine,” Pharmacological Reviews 56, no. 3 (2004): 331–349. 15317907 10.1124/pr.56.3.1

[33] A. J. Espay , P. A. LeWitt , and H. Kaufmann , “Norepinephrine Deficiency in Parkinson's Disease: The Case for Noradrenergic Enhancement,” Movement Disorders: Official Journal of the Movement Disorder Society 29, no. 14 (2014): 1710–1719. 25297066 10.1002/mds.26048

[34] E. Szabadi , “Functional Neuroanatomy of the Central Noradrenergic System,” Journal of Psychopharmacology (Oxford, England) 27, no. 8 (2013): 659–693. 23761387 10.1177/0269881113490326

[35] S. E. Loughlin , S. L. Foote , and F. E. Bloom , “Efferent Projections of Nucleus Locus Coeruleus: Topographic Organization of Cells of Origin Demonstrated by Three‐Dimensional Reconstruction,” Neuroscience 18, no. 2 (1986): 291–306. 3736860 10.1016/0306-4522(86)90155-7

[36] S. D. Robertson , N. W. Plummer , J. de Marchena , and P. Jensen , “Developmental Origins of Central Norepinephrine Neuron Diversity,” Nature Neuroscience 16, no. 8 (2013): 1016–1023. 23852112 10.1038/nn.3458 PMC4319358

[37] P. Gaspar , B. Berger , A. Febvret , A. Vigny , and J. P. Henry , “Catecholamine Innervation of the Human Cerebral Cortex as Revealed by Comparative Immunohistochemistry of Tyrosine Hydroxylase and Dopamine‐Beta‐Hydroxylase,” Journal of Comparative Neurology 279, no. 2 (1989): 249–271. 2563268 10.1002/cne.902790208

[38] J. H. Morrison , S. L. Foote , D. O'Connor , and F. E. Bloom , “Laminar, Tangential and Regional Organization of the Noradrenergic Innervation of Monkey Cortex: Dopamine‐Beta‐Hydroxylase Immunohistochemistry,” Brain Research Bulletin 9, no. 1–6 (1982): 309–319. 6756551 10.1016/0361-9230(82)90144-7

[39] D. A. Lewis and J. H. Morrison , “Noradrenergic Innervation of Monkey Prefrontal Cortex: A Dopamine‐Beta‐Hydroxylase Immunohistochemical Study,” Journal of Comparative Neurology 282, no. 3 (1989): 317–330. 2715385 10.1002/cne.902820302

[40] G. Jaim‐Etcheverry and L. M. Zieher , “DSP‐4: A Novel Compound With Neurotoxic Effects on Noradrenergic Neurons of Adult and Developing Rats,” Brain Research 188, no. 2 (1980): 513–523. 7370771 10.1016/0006-8993(80)90049-9

[41] J. H. Medina and M. L. Novas , “Parallel Changes in Brain Flunitrazepam Binding and Density of Noradrenergic Innervation,” European Journal of Pharmacology 88, no. 4 (1983): 377–382. 6134628 10.1016/0014-2999(83)90589-7

[42] K. L. Lovell , “Effects of 6‐Hydroxydopamine‐Induced Norepinephrine Depletion on Cerebellar Development,” Developmental Neuroscience 5, no. 4 (1982): 359–368. 6814890 10.1159/000112695

[43] J. R. Schank , R. Ventura , S. Puglisi‐Allegra , et al., “Dopamine Beta‐Hydroxylase Knockout Mice Have Alterations in Dopamine Signaling and Are Hypersensitive to Cocaine,” Neuropsychopharmacology: Official Publication of the American College of Neuropsychopharmacology 31, no. 10 (2006): 2221–2230. 16395294 10.1038/sj.npp.1301000

[44] A. Gheidi , C. J. Davidson , S. C. Simpson , et al., “Norepinephrine Depletion in the Brain Sex‐Dependently Modulates Aspects of Spatial Learning and Memory in Female and Male Rats,” Psychopharmacology 240, no. 12 (2023): 2585–2595. 37658879 10.1007/s00213-023-06453-0 PMC11069163

[45] L. Li , X. Feng , Z. Zhou , et al., “Stress Accelerates Defensive Responses to Looming in Mice and Involves a Locus Coeruleus‐Superior Colliculus Projection,” Current Biology 28, no. 6 (2018): 859–871.e5. 29502952 10.1016/j.cub.2018.02.005

[46] K. M. Khan , N. Balasubramanian , G. Gaudencio , et al., “Human Tau‐Overexpressing Mice Recapitulate Brainstem Involvement and Neuropsychiatric Features of Early Alzheimer's Disease,” Acta Neuropathologica Communications 11, no. 1 (2023): 57. 37009893 10.1186/s40478-023-01546-5 PMC10069039

[47] E. S. Musiek , D. D. Xiong , and D. M. Holtzman , “Sleep, Circadian Rhythms, and the Pathogenesis of Alzheimer Disease,” Experimental & Molecular Medicine 47, no. 3 (2015): e148. 25766617 10.1038/emm.2014.121 PMC4351409

[48] B. Barun , “Pathophysiological Background and Clinical Characteristics of Sleep Disorders in Multiple Sclerosis,” Clinical Neurology and Neurosurgery 115, no. Suppl 1 (2013): S82–S85. 24321163 10.1016/j.clineuro.2013.09.028

[49] B. J. Matchett , L. T. Grinberg , P. Theofilas , and M. E. Murray , “The Mechanistic Link Between Selective Vulnerability of the Locus Coeruleus and Neurodegeneration in Alzheimer's Disease,” Acta Neuropathologica 141, no. 5 (2021): 631–650. 33427939 10.1007/s00401-020-02248-1 PMC8043919

[50] A. K. Evans , E. Defensor , and M. Shamloo , “Selective Vulnerability of the Locus Coeruleus Noradrenergic System and Its Role in Modulation of Neuroinflammation, Cognition, and Neurodegeneration,” Frontiers in Pharmacology 13 (2022): 1030609. 36532725 10.3389/fphar.2022.1030609 PMC9748190

[51] H. Braak and K. Del Tredici , “The Pathological Process Underlying Alzheimer's Disease in Individuals Under Thirty,” Acta Neuropathologica 121, no. 2 (2011): 171–181. 21170538 10.1007/s00401-010-0789-4

[52] D. Sulzer , J. Bogulavsky , K. E. Larsen , et al., “Neuromelanin Biosynthesis Is Driven by Excess Cytosolic Catecholamines Not Accumulated by Synaptic Vesicles,” Proceedings of the National Academy of Sciences of the United States of America 97, no. 22 (2000): 11869–11874. 11050221 10.1073/pnas.97.22.11869 PMC17261

[53] K. S. Kendler , “Are Psychiatric Disorders Brain Diseases?‐A New Look at an Old Question,” JAMA Psychiatry 81, no. 4 (2024): 325–326. 38416478 10.1001/jamapsychiatry.2024.0036

[54] G. B. Stefano , P. Büttiker , S. Weissenberger , et al., “Artificial Intelligence: Deciphering the Links Between Psychiatric Disorders and Neurodegenerative Disease,” Brain Sciences 13, no. 7 (2023): 1055–1058. 37508987 10.3390/brainsci13071055 PMC10377467

[55] D. Veréb , M. Mijalkov , A. Canal‐Garcia , et al., “Age‐Related Differences in the Functional Topography of the Locus Coeruleus and Their Implications for Cognitive and Affective Functions,” eLife 12 (2023): 12. 10.7554/eLife.87188 PMC10471162 37650882

[56] L. Flores‐Aguilar , H. Hall , C. Orciani , et al., “Early Loss of Locus Coeruleus Innervation Promotes Cognitive and Neuropathological Changes Before Amyloid Plaque Deposition in a Transgenic Rat Model of Alzheimer's Disease,” Neuropathology and Applied Neurobiology 48, no. 6 (2022): e12835. 35822518 10.1111/nan.12835

[57] A. F. Iannitelli , M. A. Kelberman , D. J. Lustberg , et al., “The Neurotoxin DSP‐4 Dysregulates the Locus Coeruleus‐Norepinephrine System and Recapitulates Molecular and Behavioral Aspects of Prodromal Neurodegenerative Disease,” ENeuro 10, no. 1 (2023): ENEURO.0483‐22.2022. 10.1523/ENEURO.0483-22.2022 PMC9829100 36635251

[58] N. Falgàs , M. Peña‐González , A. Val‐Guardiola , et al., “Locus Coeruleus Integrity and Neuropsychiatric Symptoms in a Cohort of Early‐ and Late‐Onset Alzheimer's Disease,” Alzheimer's & Dementia: The Journal of the Alzheimer's Association 20 (2024): 6351–6364. 10.1002/alz.14131 PMC11497680 39051173

[59] M. T. Ferretti , S. Allard , V. Partridge , A. Ducatenzeiler , and A. C. Cuello , “Minocycline Corrects Early, Pre‐Plaque Neuroinflammation and Inhibits BACE‐1 in a Transgenic Model of Alzheimer's Disease‐Like Amyloid Pathology,” Journal of Neuroinflammation 9 (2012): 62. 22472085 10.1186/1742-2094-9-62 PMC3352127

[60] M. J. Dahl , M. Mather , M. Werkle‐Bergner , et al., “Locus Coeruleus Integrity Is Related to Tau Burden and Memory Loss in Autosomal‐Dominant Alzheimer's Disease,” Neurobiology of Aging 112 (2022): 39–54. 35045380 10.1016/j.neurobiolaging.2021.11.006 PMC8976827

[61] H. I. L. Jacobs , J. A. Becker , K. Kwong , et al., “In Vivo and Neuropathology Data Support Locus Coeruleus Integrity as Indicator of Alzheimer's Disease Pathology and Cognitive Decline,” Science Translational Medicine 13, no. 612 (2021): eabj2511. 34550726 10.1126/scitranslmed.abj2511 PMC8641759

[62] L. Hou , F. Sun , W. Sun , L. Zhang , and Q. Wang , “Lesion of the Locus Coeruleus Damages Learning and Memory Performance in Paraquat and Maneb‐Induced Mouse Parkinson's Disease Model,” Neuroscience 419 (2019): 129–140. 31634513 10.1016/j.neuroscience.2019.09.006

[63] C. Kjaerby , M. Andersen , N. Hauglund , et al., “Memory‐Enhancing Properties of Sleep Depend on the Oscillatory Amplitude of Norepinephrine,” Nature Neuroscience 25, no. 8 (2022): 1059–1070. 35798980 10.1038/s41593-022-01102-9 PMC9817483

[64] S. Song , Q. Wang , L. Jiang , et al., “Noradrenergic Dysfunction Accelerates LPS‐Elicited Inflammation‐Related Ascending Sequential Neurodegeneration and Deficits in Non‐motor/Motor Functions,” Brain, Behavior, and Immunity 81 (2019): 374–387. 31247288 10.1016/j.bbi.2019.06.034 PMC6754798

[65] C. Delaville , P. D. Deurwaerdère , and A. Benazzouz , “Noradrenaline and Parkinson's Disease,” Frontiers in Systems Neuroscience 5 (2011): 31. 21647359 10.3389/fnsys.2011.00031 PMC3103977

[66] T. Zaehle , I. Galazky , and K. Krauel , “The LC‐NE System as a Potential Target for Neuromodulation to Ameliorate Non‐motor Symptoms in Parkinson's Disease,” Autonomic Neuroscience: Basic & Clinical 236 (2021): 102901. 34757309 10.1016/j.autneu.2021.102901

[67] N. Pavese , M. Rivero‐Bosch , S. J. Lewis , A. L. Whone , and D. J. Brooks , “Progression of Monoaminergic Dysfunction in Parkinson's Disease: A Longitudinal 18F‐Dopa PET Study,” NeuroImage 56, no. 3 (2011): 1463–1468. 21396455 10.1016/j.neuroimage.2011.03.012

[68] M. V. Simonini , P. E. Polak , A. Sharp , S. McGuire , E. Galea , and D. L. Feinstein , “Increasing CNS Noradrenaline Reduces EAE Severity,” Journal of Neuroimmune Pharmacology: The Official Journal of the Society on NeuroImmune Pharmacology 5, no. 2 (2010): 252–259. 19957206 10.1007/s11481-009-9182-2

[69] P. E. Polak , S. Kalinin , and D. L. Feinstein , “Locus Coeruleus Damage and Noradrenaline Reductions in Multiple Sclerosis and Experimental Autoimmune Encephalomyelitis,” Brain: A Journal of Neurology 134, no. Pt 3 (2011): 665–677. 21297130 10.1093/brain/awq362 PMC3105488

[70] R. Bernard , I. A. Kerman , R. C. Thompson , et al., “Altered Expression of Glutamate Signaling, Growth Factor, and Glia Genes in the Locus Coeruleus of Patients With Major Depression,” Molecular Psychiatry 16, no. 6 (2011): 634–646. 20386568 10.1038/mp.2010.44 PMC2927798

[71] M. F. Oginsky , N. Cui , W. Zhong , C. M. Johnson , and C. Jiang , “Alterations in the Cholinergic System of Brain Stem Neurons in a Mouse Model of Rett Syndrome,” American Journal of Physiology. Cell Physiology 307, no. 6 (2014): C508–C520. 25009110 10.1152/ajpcell.00035.2014 PMC4166737

[72] Y.‐N. Yang , M. Q. Zhang , F. L. Yu , et al., “Peroxisom Proliferator‐Activated Receptor‐γ Coactivator‐1α in Neurodegenerative Disorders: A Promising Therapeutic Target,” Biochemical Pharmacology 215 (2023): 115717. 37516277 10.1016/j.bcp.2023.115717

[73] W. Bondareff , C. Q. Mountjoy , and M. Roth , “Selective Loss of Neurones of Origin of Adrenergic Projection to Cerebral Cortex (Nucleus Locus Coeruleus) in Senile Dementia,” Lancet 1, no. 8223 (1981): 783–784. 6110985 10.1016/s0140-6736(81)92657-x

[74] D. R. Thal , U. Rüb , M. Orantes , and H. Braak , “Phases of A Beta‐Deposition in the Human Brain and Its Relevance for the Development of AD,” Neurology 58, no. 12 (2002): 1791–1800. 12084879 10.1212/wnl.58.12.1791

[75] P. Theofilas , A. J. Ehrenberg , S. Dunlop , et al., “Locus Coeruleus Volume and Cell Population Changes During Alzheimer's Disease Progression: A Stereological Study in Human Postmortem Brains With Potential Implication for Early‐Stage Biomarker Discovery,” Alzheimer's & Dementia: The Journal of the Alzheimer's Association 13, no. 3 (2017): 236–246. 10.1016/j.jalz.2016.06.2362 PMC5298942 27513978

[76] J. Oh , R. A. Eser , A. J. Ehrenberg , et al., “Profound Degeneration of Wake‐Promoting Neurons in Alzheimer's Disease,” Alzheimer's & Dementia: The Journal of the Alzheimer's Association 15, no. 10 (2019): 1253–1263. 10.1016/j.jalz.2019.06.3916 PMC6801040 31416793

[77] R. Beardmore , R. Hou , A. Darekar , et al., “The Locus Coeruleus in Aging and Alzheimer's Disease: A Postmortem and Brain Imaging Review,” Journal of Alzheimer's Disease: JAD 83, no. 1 (2021): 5–22. 34219717 10.3233/JAD-210191 PMC8461706

[78] Y. Chen , T. Chen , and R. Hou , “Locus Coeruleus in the Pathogenesis of Alzheimer's Disease: A Systematic Review,” Alzheimer's & Dementia 8, no. 1 (2022): e12257. 10.1002/trc2.12257 PMC8900465 35282658

[79] C.‐P. Lin , I. Frigerio , J. G. J. M. Bol , et al., “Microstructural Integrity of the Locus Coeruleus and Its Tracts Reflect Noradrenergic Degeneration in Alzheimer's Disease and Parkinson's Disease,” Translational Neurodegeneration 13, no. 1 (2024): 9. 38336865 10.1186/s40035-024-00400-5 PMC10854137

[80] F. E. McAlpine , J. K. Lee , A. S. Harms , et al., “Inhibition of Soluble TNF Signaling in a Mouse Model of Alzheimer's Disease Prevents Pre‐Plaque Amyloid‐Associated Neuropathology,” Neurobiology of Disease 34, no. 1 (2009): 163–177. 19320056 10.1016/j.nbd.2009.01.006 PMC2948857

[81] L. Kelly , M. Seifi , R. Ma , et al., “Identification of Intraneuronal Amyloid Beta Oligomers in Locus Coeruleus Neurons of Alzheimer's Patients and Their Potential Impact on Inhibitory Neurotransmitter Receptors and Neuronal Excitability,” Neuropathology and Applied Neurobiology 47, no. 4 (2021): 488–505. 33119191 10.1111/nan.12674

[82] S. S. Kang , X. Liu , E. H. Ahn , et al., “Norepinephrine Metabolite DOPEGAL Activates AEP and Pathological Tau Aggregation in Locus Coeruleus,” Journal of Clinical Investigation 130, no. 1 (2020): 422–437. 31793911 10.1172/JCI130513 PMC6934194

[83] F. S. Giorgi , F. Biagioni , A. Galgani , N. Pavese , G. Lazzeri , and F. Fornai , “Locus Coeruleus Modulates Neuroinflammation in Parkinsonism and Dementia,” International Journal of Molecular Sciences 21, no. 22 (2020): 8630–8650. 33207731 10.3390/ijms21228630 PMC7697920

[84] O. Baytas , J. A. Kauer , and E. M. Morrow , “Loss of Mitochondrial Enzyme GPT2 Causes Early Neurodegeneration in Locus Coeruleus,” Neurobiology of Disease 173 (2022): 105831. 35908744 10.1016/j.nbd.2022.105831 PMC9669404

[85] S. S. Kang , E. H. Ahn , X. Liu , et al., “ApoE4 Inhibition of VMAT2 in the Locus Coeruleus Exacerbates Tau Pathology in Alzheimer's Disease,” Acta Neuropathologica 142, no. 1 (2021): 139–158. 33895869 10.1007/s00401-021-02315-1 PMC8217363

[86] M. P. Kummer , T. Hammerschmidt , A. Martinez , et al., “Ear2 Deletion Causes Early Memory and Learning Deficits in APP/PS1 Mice,” Journal of Neuroscience: The Official Journal of the Society for Neuroscience 34, no. 26 (2014): 8845–8854. 24966384 10.1523/JNEUROSCI.4027-13.2014 PMC4147626

[87] K. S. Rommelfanger and D. Weinshenker , “Norepinephrine: The Redheaded Stepchild of Parkinson's Disease,” Biochemical Pharmacology 74, no. 2 (2007): 177–190. 17416354 10.1016/j.bcp.2007.01.036

[88] R. R. Wyrofsky , B. A. S. Reyes , X. Y. Zhang , S. Bhatnagar , L. G. Kirby , and E. J. van Bockstaele , “Endocannabinoids, Stress Signaling, and the Locus Coeruleus‐Norepinephrine System,” Neurobiology of Stress 11 (2019): 100176. 31236436 10.1016/j.ynstr.2019.100176 PMC6582240

[89] L. A. Matschke , M. A. Komadowski , A. Stöhr , et al., “Enhanced Firing of Locus Coeruleus Neurons and SK Channel Dysfunction Are Conserved in Distinct Models of Prodromal Parkinson's Disease,” Scientific Reports 12, no. 1 (2022): 3180. 35210472 10.1038/s41598-022-06832-1 PMC8873463

[90] L. M. Butkovich , M. C. Houser , T. Chalermpalanupap , et al., “Transgenic Mice Expressing Human α‐Synuclein in Noradrenergic Neurons Develop Locus Ceruleus Pathology and Nonmotor Features of Parkinson's Disease,” Journal of Neuroscience: The Official Journal of the Society for Neuroscience 40, no. 39 (2020): 7559–7576. 32868457 10.1523/JNEUROSCI.1468-19.2020 PMC7511194

[91] A. F. Iannitelli , L. Hassenein , B. Mulvey , et al., Tyrosinase‐Induced Neuromelanin Accumulation Triggers Rapid Dysregulation and Degeneration of the Mouse Locus Coeruleus (England: BioRxiv: The Preprint Server For Biology, 2023).

[92] K. Cui , F. Yang , T. Tufan , et al., “Restoration of Noradrenergic Function in Parkinson's Disease Model Mice,” ASN Neuro 13 (2021): 17590914211009730. 33940943 10.1177/17590914211009730 PMC8114769

[93] Y. Zhan , M. U. Raza , L. Yuan , and M. Y. Zhu , “Critical Role of Oxidatively Damaged DNA in Selective Noradrenergic Vulnerability,” Neuroscience 422 (2019): 184–201. 31698021 10.1016/j.neuroscience.2019.09.036 PMC9338788

[94] B. Huynh , Y. Fu , D. Kirik , J. M. Shine , and G. M. Halliday , “Comparison of Locus Coeruleus Pathology With Nigral and Forebrain Pathology in Parkinson's Disease,” Movement Disorders: Official Journal of the Movement Disorder Society 36, no. 9 (2021): 2085–2093. 33899954 10.1002/mds.28615

[95] Y. Mukai , T. S. Okubo , M. Lazarus , D. Ono , K. F. Tanaka , and A. Yamanaka , “Prostaglandin E2 Induces Long‐Lasting Inhibition of Noradrenergic Neurons in the Locus Coeruleus and Moderates the Behavioral Response to Stressors,” Journal of Neuroscience: The Official Journal of the Society for Neuroscience 43, no. 47 (2023): 7982–7999. 37734949 10.1523/JNEUROSCI.0353-23.2023 PMC10669809

[96] O. Borodovitsyna , B. C. Duffy , A. E. Pickering , and D. J. Chandler , “Anatomically and Functionally Distinct Locus Coeruleus Efferents Mediate Opposing Effects on Anxiety‐Like Behavior,” Neurobiology of Stress 13 (2020): 100284. 33344735 10.1016/j.ynstr.2020.100284 PMC7739179

[97] M. Privitera , L. M. von Ziegler , A. Floriou‐Servou , et al., “Noradrenaline Release From the Locus Coeruleus Shapes Stress‐Induced Hippocampal Gene Expression,” eLife 12 (2024): 12. 10.7554/eLife.88559 PMC10937036 38477670

[98] C. Walton , R. King , L. Rechtman , et al., “Rising Prevalence of Multiple Sclerosis Worldwide: Insights From the Atlas of MS,” in Multiple Sclerosis, vol. 26, 3rd ed. (Houndmills, Basingstoke, England: SAGE Publications Ltd., 2020), 1816–1821. 33174475 10.1177/1352458520970841 PMC7720355

[99] D. S. Reich , C. F. Lucchinetti , and P. A. Calabresi , “Multiple Sclerosis,” New England Journal of Medicine 378, no. 2 (2018): 169–180. 29320652 10.1056/NEJMra1401483 PMC6942519

[100] T. Carandini , M. Mancini , I. Bogdan , et al., “In Vivo Evidence of Functional Disconnection Between Brainstem Monoaminergic Nuclei and Brain Networks in Multiple Sclerosis,” Multiple Sclerosis and Related Disorders 56 (2021): 103224. 34461571 10.1016/j.msard.2021.103224

[101] A. Carotenuto , H. Wilson , B. Giordano , et al., “Impaired Connectivity Within Neuromodulatory Networks in Multiple Sclerosis and Clinical Implications,” Journal of Neurology 267, no. 7 (2020): 2042–2053. 32219555 10.1007/s00415-020-09806-3 PMC7320961

[102] A. E. Kirkland , M. C. Fadus , S. A. Gruber , K. M. Gray , T. E. Wilens , and L. M. Squeglia , “A Scoping Review of the Use of Cannabidiol in Psychiatric Disorders,” Psychiatry Research 308 (2022): 114347. 34952255 10.1016/j.psychres.2021.114347 PMC8799523

[103] D. Weinshenker , “Long Road to Ruin: Noradrenergic Dysfunction in Neurodegenerative Disease,” Trends in Neurosciences 41, no. 4 (2018): 211–223. 29475564 10.1016/j.tins.2018.01.010 PMC5878728

[104] T. James , B. Kula , S. Choi , S. S. Khan , L. K. Bekar , and N. A. Smith , “Locus Coeruleus in Memory Formation and Alzheimer's Disease,” European Journal of Neuroscience 54, no. 8 (2021): 6948–6959. 33190318 10.1111/ejn.15045 PMC8121900

[105] J. G. McCall , R. al‐Hasani , E. R. Siuda , et al., “CRH Engagement of the Locus Coeruleus Noradrenergic System Mediates Stress‐Induced Anxiety,” Neuron 87, no. 3 (2015): 605–620. 26212712 10.1016/j.neuron.2015.07.002 PMC4529361

[106] R. P. Tillage , S. L. Foster , D. Lustberg , L. C. Liles , K. E. McCann , and D. Weinshenker , “Co‐Released Norepinephrine and Galanin Act on Different Timescales to Promote Stress‐Induced Anxiety‐Like Behavior,” Neuropsychopharmacology: Official Publication of the American College of Neuropsychopharmacology 46, no. 8 (2021): 1535–1543. 33911187 10.1038/s41386-021-01011-8 PMC8208976

[107] J. G. McCall , E. R. Siuda , D. L. Bhatti , et al., “Locus Coeruleus to Basolateral Amygdala Noradrenergic Projections Promote Anxiety‐Like Behavior,” eLife 6 (2017): 6. 10.7554/eLife.18247 PMC5550275 28708061

[108] S. Liu , A. Abdellaoui , K. J. H. Verweij , and G. A. van Wingen , “Gene Expression Has Distinct Associations With Brain Structure and Function in Major Depressive Disorder. Advanced Science,” Advanced Science 10, no. 7 (2023): e2205486. 36638259 10.1002/advs.202205486 PMC9982587

[109] K. Seki , S. Yoshida , and M. K. Jaiswal , “Molecular Mechanism of Noradrenaline During the Stress‐Induced Major Depressive Disorder,” Neural Regeneration Research 13, no. 7 (2018): 1159–1169. 30028316 10.4103/1673-5374.235019 PMC6065220

[110] A. Guinea‐Izquierdo , M. Giménez , I. Martínez‐Zalacaín , et al., “Lower Locus Coeruleus MRI Intensity in Patients With Late‐Life Major Depression,” PeerJ 9 (2021): e10828. 33628639 10.7717/peerj.10828 PMC7894108

[111] L. Jacobson , “Glucocorticoid Receptor Deletion From Locus Coeruleus Norepinephrine Neurons Promotes Depression‐Like Social Withdrawal in Female but Not Male Mice,” Brain Research 1710 (2019): 82–91. 30576626 10.1016/j.brainres.2018.12.026

[112] S. Zhang , C. M. Johnson , N. Cui , et al., “An Optogenetic Mouse Model of Rett Syndrome Targeting on Catecholaminergic Neurons,” Journal of Neuroscience Research 94, no. 10 (2016): 896–906. 27317352 10.1002/jnr.23760 PMC4990462

[113] J. A. Ross , P. McGonigle , and E. J. Van Bockstaele , “Locus Coeruleus, Norepinephrine and Aβ Peptides in Alzheimer's Disease,” Neurobiology of Stress 2 (2015): 73–84. 26618188 10.1016/j.ynstr.2015.09.002 PMC4657149

[114] S. Makino , M. A. Smith , and P. W. Gold , “Regulatory Role of Glucocorticoids and Glucocorticoid Receptor mRNA Levels on Tyrosine Hydroxylase Gene Expression in the Locus Coeruleus During Repeated Immobilization Stress,” Brain Research 943, no. 2 (2002): 216–223. 12101044 10.1016/s0006-8993(02)02647-1

[115] D. C. German , K. F. Manaye , C. L. White, III , et al., “Disease‐Specific Patterns of Locus Coeruleus Cell Loss,” Annals of Neurology 32, no. 5 (1992): 667–676. 1449247 10.1002/ana.410320510

[116] I. L. Gutiérrez , M. González‐Prieto , J. R. Caso , B. García‐Bueno , J. C. Leza , and J. L. M. Madrigal , “Reboxetine Treatment Reduces Neuroinflammation and Neurodegeneration in the 5xFAD Mouse Model of Alzheimer's Disease: Role of CCL2,” Molecular Neurobiology 56, no. 12 (2019): 8628–8642. 31297718 10.1007/s12035-019-01695-6

[117] A. I. Levey , D. Qiu , L. Zhao , et al., “A Phase II Study Repurposing Atomoxetine for Neuroprotection in Mild Cognitive Impairment,” Brain: A Journal of Neurology 145, no. 6 (2022): 1924–1938. 34919634 10.1093/brain/awab452 PMC9630662

[118] H. Tsunekawa , Y. Noda , A. Mouri , F. Yoneda , and T. Nabeshima , “Synergistic Effects of Selegiline and Donepezil on Cognitive Impairment Induced by Amyloid Beta (25‐35),” Behavioural Brain Research 190, no. 2 (2008): 224–232. 18420288 10.1016/j.bbr.2008.03.002

[119] D. Braun , J. L. M. Madrigal , and D. L. Feinstein , “Noradrenergic Regulation of Glial Activation: Molecular Mechanisms and Therapeutic Implications,” Current Neuropharmacology 12, no. 4 (2014): 342–352. 25342942 10.2174/1570159X12666140828220938 PMC4207074

[120] D. Braun and D. L. Feinstein , “The Locus Coeruleus Neuroprotective Drug Vindeburnol Normalizes Behavior in the 5xFAD Transgenic Mouse Model of Alzheimer's Disease,” Brain Research 1702 (2019): 29–37. 29274883 10.1016/j.brainres.2017.12.028

[121] G. Kreiner , K. Rafa‐Zabłocka , J. Barut , et al., “Stimulation of Noradrenergic Transmission by Reboxetine Is Beneficial for a Mouse Model of Progressive Parkinsonism,” Scientific Reports 9, no. 1 (2019): 5262. 30918302 10.1038/s41598-019-41756-3 PMC6437187

[122] R. Pacheco , F. Contreras , and M. Zouali , “The Dopaminergic System in Autoimmune Diseases,” Frontiers in Immunology 5 (2014): 117. 24711809 10.3389/fimmu.2014.00117 PMC3968755

[123] A. Torrillas‐de la Cal , S. Torres‐Sanchez , L. Bravo , et al., “Chemogenetic Activation of Locus Coeruleus Neurons Ameliorates the Severity of Multiple Sclerosis,” Journal of Neuroinflammation 20, no. 1 (2023): 198. 37658434 10.1186/s12974-023-02865-z PMC10474779

[124] A. Mendiguren , E. Aostri , I. Rodilla , I. Pujana , E. Noskova , and J. Pineda , “Cannabigerol Modulates α2‐Adrenoceptor and 5‐HT1A Receptor‐Mediated Electrophysiological Effects on Dorsal Raphe Nucleus and Locus Coeruleus Neurons and Anxiety Behavior in Rat,” Frontiers in Pharmacology 14 (2023): 1183019. 37305529 10.3389/fphar.2023.1183019 PMC10249961

[125] Y. Fan , P. Chen , M. U. Raza , et al., “Altered Expression of Phox2 Transcription Factors in the Locus Coeruleus in Major Depressive Disorder Mimicked by Chronic Stress and Corticosterone Treatment in Vivo and in Vitro,” Neuroscience 393 (2018): 123–137. 30315878 10.1016/j.neuroscience.2018.09.038 PMC6246807

[126] D. Tse , L. Privitera , A. C. Norton , et al., “Cell‐Type‐Specific Optogenetic Stimulation of the Locus Coeruleus Induces Slow‐Onset Potentiation and Enhances Everyday Memory in Rats,” Proceedings of the National Academy of Sciences of the United States of America 120, no. 46 (2023): e2307275120. 37931094 10.1073/pnas.2307275120 PMC10655220

[127] C. Sagheddu , P. Devoto , S. Aroni , P. Saba , M. Pistis , and G. L. Gessa , “Combined α2‐ and D2‐Receptor Blockade Activates Noradrenergic and Dopaminergic Neurons, but Extracellular Dopamine in the Prefrontal Cortex Is Determined by Uptake and Release From Noradrenergic Terminals,” Frontiers in Pharmacology 14 (2023): 1238115. 37680715 10.3389/fphar.2023.1238115 PMC10482411

[128] P. Jovanovic , Y. Wang , J. P. Vit , et al., “Sustained Chemogenetic Activation of Locus Coeruleus Norepinephrine Neurons Promotes Dopaminergic Neuron Survival in Synucleinopathy,” PLoS One 17, no. 3 (2022): e0263074. 35316276 10.1371/journal.pone.0263074 PMC8939823

[129] D. C. Matthews , A. Ritter , R. G. Thomas , et al., “Rasagiline Effects on Glucose Metabolism, Cognition, and Tau in Alzheimer's Dementia,” Alzheimer's & Dementia 7, no. 1 (2021): e12106. 10.1002/trc2.12106 PMC7882538 33614888

[130] S.‐X. Cao , Y. Zhang , X. Y. Hu , et al., “ErbB4 Deletion in Noradrenergic Neurons in the Locus Coeruleus Induces Mania‐Like Behavior via Elevated Catecholamines,” eLife 7 (2018): 7. 10.7554/eLife.39907 PMC6185106 30179154

[131] Y.‐P. Xu , X. Y. Cui , Y. T. Liu , S. Y. Cui , and Y. H. Zhang , “Ginsenoside Rg1 Promotes Sleep in Rats by Modulating the Noradrenergic System in the Locus Coeruleus and Serotonergic System in the Dorsal Raphe Nucleus,” Biomedicine & Pharmacotherapy 116 (2019): 109009. 31154268 10.1016/j.biopha.2019.109009

[132] Q. Zhang , Y. Xue , K. Wei , et al., “Locus Coeruleus‐Dorsolateral Septum Projections Modulate Depression‐Like Behaviors via BDNF but Not Norepinephrine. Advanced Science,” Advanced Science 11, no. 10 (2024): e2303503. 38155473 10.1002/advs.202303503 PMC10933643

[133] P. Follesa , F. Biggio , G. Gorini , et al., “Vagus Nerve Stimulation Increases Norepinephrine Concentration and the Gene Expression of BDNF and bFGF in the Rat Brain,” Brain Research 1179 (2007): 28–34. 17920573 10.1016/j.brainres.2007.08.045

[134] R. Fukabori , Y. Iguchi , S. Kato , et al., “Enhanced Retrieval of Taste Associative Memory by Chemogenetic Activation of Locus Coeruleus Norepinephrine Neurons,” Journal of Neuroscience: The Official Journal of the Society for Neuroscience 40, no. 43 (2020): 8367–8385. 32994339 10.1523/JNEUROSCI.1720-20.2020 PMC7577602

[135] H. Antila , I. Kwak , A. Choi , et al., “A Noradrenergic‐Hypothalamic Neural Substrate for Stress‐Induced Sleep Disturbances,” Proceedings of the National Academy of Sciences of the United States of America 119, no. 45 (2022): e2123528119. 36331996 10.1073/pnas.2123528119 PMC9659376

[136] J. A. Thomson , J. Itskovitz‐Eldor , S. S. Shapiro , et al., “Embryonic Stem Cell Lines Derived From Human Blastocysts,” Science 282, no. 5391 (1998): 1145–1147. 9804556 10.1126/science.282.5391.1145

[137] K. Takahashi , K. Tanabe , M. Ohnuki , et al., “Induction of Pluripotent Stem Cells From Adult Human Fibroblasts by Defined Factors,” Cell 131, no. 5 (2007): 861–872. 18035408 10.1016/j.cell.2007.11.019

[138] D. A. Ogi and S. Jin , “Transcriptome‐Powered Pluripotent Stem Cell Differentiation for Regenerative Medicine,” Cells 12, no. 10 (2023): 1–4. 10.3390/cells12101442 PMC10217280 37408278

[139] S. Shrestha , N. C. Anderson , L. B. Grabel , J. R. Naegele , and G. B. Aaron , “Development of Electrophysiological and Morphological Properties of Human Embryonic Stem Cell‐Derived GABAergic Interneurons at Different Times After Transplantation Into the Mouse Hippocampus,” PLoS One 15, no. 8 (2020): e0237426. 32813731 10.1371/journal.pone.0237426 PMC7444508

[140] C.‐Y. Chang , H. C. Ting , C. A. Liu , et al., “Induced Pluripotent Stem Cell (iPSC)‐based Neurodegenerative Disease Models for Phenotype Recapitulation and Drug Screening,” Molecules 25, no. 8 (2020): 2000–2020. 32344649 10.3390/molecules25082000 PMC7221979

[141] Z. Zhang , H. Sheng , L. Liao , et al., “Mesenchymal Stem Cell‐Conditioned Medium Improves Mitochondrial Dysfunction and Suppresses Apoptosis in Okadaic Acid‐Treated SH‐SY5Y Cells by Extracellular Vesicle Mitochondrial Transfer,” Journal of Alzheimer's Disease: JAD 78, no. 3 (2020): 1161–1176. 33104031 10.3233/JAD-200686

[142] M.‐Y. Cha , Y. W. Kwon , H. S. Ahn , et al., “Protein‐Induced Pluripotent Stem Cells Ameliorate Cognitive Dysfunction and Reduce Aβ Deposition in a Mouse Model of Alzheimer's Disease,” Stem Cells Translational Medicine 6, no. 1 (2017): 293–305. 28170178 10.5966/sctm.2016-0081 PMC5442740

[143] T. Zhang , W. Ke , X. Zhou , et al., “Human Neural Stem Cells Reinforce Hippocampal Synaptic Network and Rescue Cognitive Deficits in a Mouse Model of Alzheimer's Disease,” Stem Cell Reports 13, no. 6 (2019): 1022–1037. 31761676 10.1016/j.stemcr.2019.10.012 PMC6915849

[144] Z.‐B. Wang , Z. T. Wang , Y. Sun , L. Tan , and J. T. Yu , “The Future of Stem Cell Therapies of Alzheimer's Disease,” Ageing Research Reviews 80 (2022): 101655. 35660003 10.1016/j.arr.2022.101655

[145] R. van der Kant , V. F. Langness , C. M. Herrera , et al., “Cholesterol Metabolism Is a Druggable Axis That Independently Regulates Tau and Amyloid‐β in iPSC‐Derived Alzheimer's Disease Neurons,” Cell Stem Cell 24, no. 3 (2019): 363–375.e9. 30686764 10.1016/j.stem.2018.12.013 PMC6414424

[146] S. Kriks , J. W. Shim , J. Piao , et al., “Dopamine Neurons Derived From Human ES Cells Efficiently Engraft in Animal Models of Parkinson's Disease,” Nature 480, no. 7378 (2011): 547–551. 22056989 10.1038/nature10648 PMC3245796

[147] Z. Chen and G. Zhao , “First‐In‐Human Transplantation of Autologous Induced Neural Stem Cell‐Derived Dopaminergic Precursors to Treat Parkinson's Disease,” Science Bulletin 68, no. 22 (2023): 2700–2703. 37919161 10.1016/j.scib.2023.10.020

[148] S. Jiang , H. Wang , C. Yang , et al., “Phase 1 Study of Safety and Preliminary Efficacy of Intranasal Transplantation of Human Neural Stem Cells (ANGE‐S003) in Parkinson's Disease,” Journal of Neurology, Neurosurgery, and Psychiatry 95 (2024): 1102–1111. 38724232 10.1136/jnnp-2023-332921

[149] Y. Xin , J. Gao , R. Hu , et al., “Changes of Immune Parameters of T Lymphocytes and Macrophages in EAE Mice After BM‐MSCs Transplantation,” Immunology Letters 225 (2020): 66–73. 32544469 10.1016/j.imlet.2020.05.005

[150] A. Genchi , E. Brambilla , F. Sangalli , et al., “Neural Stem Cell Transplantation in Patients With Progressive Multiple Sclerosis: An Open‐Label, Phase 1 Study,” Nature Medicine 29, no. 1 (2023): 75–85. 10.1038/s41591-022-02097-3 PMC9873560 36624312

[151] G. Boffa , A. Signori , L. Massacesi , et al., “Hematopoietic Stem Cell Transplantation in People With Active Secondary Progressive Multiple Sclerosis,” Neurology 100, no. 11 (2023): e1109–e1122. 36543569 10.1212/WNL.0000000000206750 PMC10074454

[152] O. Lykhmus , L. Koval , L. Voytenko , et al., “Intravenously Injected Mesenchymal Stem Cells Penetrate the Brain and Treat Inflammation‐Induced Brain Damage and Memory Impairment in Mice,” Frontiers in Pharmacology 10 (2019): 355. 31057400 10.3389/fphar.2019.00355 PMC6479176

[153] T. Yin , Y. Liu , W. Ji , et al., “Engineered Mesenchymal Stem Cell‐Derived Extracellular Vesicles: A State‐Of‐The‐Art Multifunctional Weapon Against Alzheimer's Disease,” Theranostics 13, no. 4 (2023): 1264–1285. 36923533 10.7150/thno.81860 PMC10008732

[154] M. Losurdo , M. Pedrazzoli , C. D'Agostino , et al., “Intranasal Delivery of Mesenchymal Stem Cell‐Derived Extracellular Vesicles Exerts Immunomodulatory and Neuroprotective Effects in a 3xTg Model of Alzheimer's Disease,” Stem Cells Translational Medicine 9, no. 9 (2020): 1068–1084. 32496649 10.1002/sctm.19-0327 PMC7445021

[155] Y. Yoo , G. Neumayer , Y. Shibuya , M. M. D. Mader , and M. Wernig , “A Cell Therapy Approach to Restore Microglial Trem2 Function in a Mouse Model of Alzheimer's Disease,” Cell Stem Cell 30, no. 8 (2023): 1392. 37802040 10.1016/j.stem.2023.08.011

[156] A. Kirkeby , J. Nelander , D. B. Hoban , et al., “Preclinical Quality, Safety, and Efficacy of a Human Embryonic Stem Cell‐Derived Product for the Treatment of Parkinson's Disease, STEM‐PD,” Cell Stem Cell 30, no. 10 (2023): 1299–1314.e9. 37802036 10.1016/j.stem.2023.08.014

[157] A. Bose , G. A. Petsko , and L. Studer , “Induced Pluripotent Stem Cells: A Tool for Modeling Parkinson's Disease,” Trends in Neurosciences 45, no. 8 (2022): 608–620. 35667922 10.1016/j.tins.2022.05.001 PMC9576003

[158] K.‐C. Sonntag , B. Song , N. Lee , et al., “Pluripotent Stem Cell‐Based Therapy for Parkinson's Disease: Current Status and Future Prospects,” Progress in Neurobiology 168 (2018): 1–20. 29653250 10.1016/j.pneurobio.2018.04.005 PMC6077089

[159] M. Parmar , S. Grealish , and C. Henchcliffe , “The Future of Stem Cell Therapies for Parkinson Disease,” Nature Reviews. Neuroscience 21, no. 2 (2020): 103–115. 31907406 10.1038/s41583-019-0257-7

[160] T.‐Y. Park , J. Jeon , N. Lee , et al., “Co‐Transplantation of Autologous Treg Cells in a Cell Therapy for Parkinson's Disease,” Nature 619, no. 7970 (2023): 606–615. 37438521 10.1038/s41586-023-06300-4 PMC12012854

[161] T. W. Kim , S. Y. Koo , M. Riessland , et al., “TNF‐NF‐κB‐p53 Axis Restricts In Vivo Survival of hPSC‐Derived Dopamine Neurons,” Cell 187 (2024): 3671–3689.e23. 38866017 10.1016/j.cell.2024.05.030 PMC11641762

[162] Y. Tao , S. C. Vermilyea , M. Zammit , et al., “Autologous Transplant Therapy Alleviates Motor and Depressive Behaviors in Parkinsonian Monkeys,” Nature Medicine 27, no. 4 (2021): 632–639. 10.1038/s41591-021-01257-1 PMC8198752 33649496

[163] J. S. Schweitzer , B. Song , T. M. Herrington , et al., “Personalized iPSC‐Derived Dopamine Progenitor Cells for Parkinson's Disease,” New England Journal of Medicine 382, no. 20 (2020): 1926–1932. 32402162 10.1056/NEJMoa1915872 PMC7288982

[164] P. Xu , H. He , Q. Gao , et al., “Human Midbrain Dopaminergic Neuronal Differentiation Markers Predict Cell Therapy Outcomes in a Parkinson's Disease Model,” Journal of Clinical Investigation 132, no. 14 (2022): e156768. 35700056 10.1172/JCI156768 PMC9282930

[165] W. Dong , S. Liu , S. Li , and Z. Wang , “Cell Reprogramming Therapy for Parkinson's Disease,” Neural Regeneration Research 19, no. 11 (2024): 2444–2455. 38526281 10.4103/1673-5374.390965 PMC11090434

[166] P. Pinjala , K. P. Tryphena , R. Prasad , et al., “CRISPR/Cas9 Assisted Stem Cell Therapy in Parkinson's Disease,” Biomaterials Research 27, no. 1 (2023): 46. 37194005 10.1186/s40824-023-00381-y PMC10190035

[167] J. S. Graves , K. M. Krysko , L. H. Hua , M. Absinta , R. J. M. Franklin , and B. M. Segal , “Ageing and Multiple Sclerosis,” Lancet Neurology 22, no. 1 (2023): 66–77. 36216015 10.1016/S1474-4422(22)00184-3

[168] T. Kalincik , S. Sharmin , I. Roos , et al., “Comparative Effectiveness of Autologous Hematopoietic Stem Cell Transplant vs Fingolimod, Natalizumab, and Ocrelizumab in Highly Active Relapsing‐Remitting Multiple Sclerosis,” JAMA Neurology 80, no. 7 (2023): 702–713. 37437240 10.1001/jamaneurol.2023.1184 PMC10186210

[169] L. Feng , J. Chao , P. Ye , et al., “Developing Hypoimmunogenic Human iPSC‐Derived Oligodendrocyte Progenitor Cells as an off‐The‐Shelf Cell Therapy for Myelin Disorders,” Advanced Science 10, no. 23 (2023): e2206910. 37271923 10.1002/advs.202206910 PMC10427412

[170] Z. Wang , L. Zhang , Y. Yang , et al., “Oligodendrocyte Progenitor Cell Transplantation Ameliorates Preterm Infant Cerebral White Matter Injury in Rats Model,” Neuropsychiatric Disease and Treatment 19 (2023): 1935–1947. 37719062 10.2147/NDT.S414493 PMC10503552

[171] J. Xu , J. Zhao , R. Wang , et al., “Shh and Olig2 Sequentially Regulate Oligodendrocyte Differentiation From hiPSCs for the Treatment of Ischemic Stroke,” Theranostics 12, no. 7 (2022): 3131–3149. 35547747 10.7150/thno.69217 PMC9065175

[172] V. Fossati , L. Peruzzotti‐Jametti , and S. Pluchino , “A neural stem‐cell treatment for progressive multiple sclerosis,” Nature Medicine 29, no. 1 (2023): 27–28. 10.1038/s41591-022-02164-9 36639562

[173] J. A. Smith , A. M. Nicaise , R. B. Ionescu , R. Hamel , L. Peruzzotti‐Jametti , and S. Pluchino , “Stem Cell Therapies for Progressive Multiple Sclerosis,” Frontiers in Cell and Developmental Biology 9 (2021): 696434. 34307372 10.3389/fcell.2021.696434 PMC8299560

[174] S. Ramalingam and A. Shah , “Stem Cell Therapy as a Treatment for Autoimmune Disease‐Updates in Lupus, Scleroderma, and Multiple Sclerosis,” Current Allergy and Asthma Reports 21, no. 3 (2021): 22. 33759038 10.1007/s11882-021-00996-y

[175] P. Aroca , B. Lorente‐Cánovas , F. R. Mateos , and L. Puelles , “Locus Coeruleus Neurons Originate in Alar Rhombomere 1 and Migrate Into the Basal Plate: Studies in Chick and Mouse Embryos,” Journal of Comparative Neurology 496, no. 6 (2006): 802–818. 16628617 10.1002/cne.20957

[176] C. Watson , T. Shimogori , and L. Puelles , “Mouse Fgf8‐Cre‐LacZ Lineage Analysis Defines the Territory of the Postnatal Mammalian Isthmus,” Journal of Comparative Neurology 525, no. 12 (2017): 2782–2799. 28510270 10.1002/cne.24242

[177] A. A. Stepanenko and V. M. Kavsan , “Immortalization and Malignant Transformation of Eukaryotic Cells,” Tsitologiia i Genetika 46, no. 2 (2012): 36–75. 22679821

[178] L. Panman , E. Andersson , Z. Alekseenko , et al., “Transcription Factor‐Induced Lineage Selection of Stem‐Cell‐Derived Neural Progenitor Cells,” Cell Stem Cell 8, no. 6 (2011): 663–675. 21624811 10.1016/j.stem.2011.04.001

[179] J. Mong , L. Panman , Z. Alekseenko , et al., “Transcription Factor‐Induced Lineage Programming of Noradrenaline and Motor Neurons From Embryonic Stem Cells,” Stem Cells 32, no. 3 (2014): 609–622. 24549637 10.1002/stem.1585

[180] P. C. Holm , F. J. Rodríguez , J. Kele , G. Castelo‐Branco , J. Kitajewski , and E. Arenas , “BMPs, FGF8 and Wnts Regulate the Differentiation of Locus Coeruleus Noradrenergic Neuronal Precursors,” Journal of Neurochemistry 99, no. 1 (2006): 343–352. 16987254 10.1111/j.1471-4159.2006.04039.x

[181] Y. Tao , X. Li , Q. Dong , et al., “Generation of Locus Coeruleus Norepinephrine Neurons From Human Pluripotent Stem Cells,” Nature Biotechnology 42 (2023): 1404–1416. 10.1038/s41587-023-01977-4 PMC11392812 37974010

[182] P. Choudhary , A. G. Pacholko , J. Palaschuk , and L. K. Bekar , “The Locus Coeruleus Neurotoxin, DSP4, and/or a High Sugar Diet Induce Behavioral and Biochemical Alterations in Wild‐Type Mice Consistent With Alzheimers Related Pathology,” Metabolic Brain Disease 33, no. 5 (2018): 1563–1571. 29862455 10.1007/s11011-018-0263-x

[183] M. Xiong , Y. Tao , Q. Gao , et al., “Human Stem Cell‐Derived Neurons Repair Circuits and Restore Neural Function,” Cell Stem Cell 28, no. 1 (2021): 112–126.e6. 32966778 10.1016/j.stem.2020.08.014 PMC7796915

[184] T. J. Collier , D. M. Gash , and J. R. Sladek , “Transplantation of Norepinephrine Neurons Into Aged Rats Improves Performance of a Learned Task,” Brain Research 448, no. 1 (1988): 77–87. 3390719 10.1016/0006-8993(88)91103-1

[185] E. M. Quintero , L. M. Willis , V. Zaman , et al., “Glial Cell Line‐Derived Neurotrophic Factor Is Essential for Neuronal Survival in the Locus Coeruleus‐Hippocampal Noradrenergic Pathway,” Neuroscience 124, no. 1 (2004): 137–146. 14960346 10.1016/j.neuroscience.2003.11.001

[186] N. W. Plummer , E. L. Scappini , K. G. Smith , C. J. Tucker , and P. Jensen , “Two Subpopulations of Noradrenergic Neurons in the Locus Coeruleus Complex Distinguished by Expression of the Dorsal Neural Tube Marker Pax7,” Frontiers in Neuroanatomy 11 (2017): 60. 28775681 10.3389/fnana.2017.00060 PMC5518464

[187] Y. Tao and S.‐C. Zhang , “Neural Subtype Specification From Human Pluripotent Stem Cells,” Cell Stem Cell 19, no. 5 (2016): 573–586. 27814479 10.1016/j.stem.2016.10.015 PMC5127287

[188] S. Ghosh and J. H. R. Maunsell , “Locus Coeruleus Norepinephrine Contributes to Visual‐Spatial Attention by Selectively Enhancing Perceptual Sensitivity,” Neuron 112, no. 13 (2024): 2231–2240.e5. 38701788 10.1016/j.neuron.2024.04.001 PMC11223979

[189] B. L. Tang , “Axon Regeneration Induced by Environmental Enrichment‐ Epigenetic Mechanisms,” Neural Regeneration Research 15, no. 1 (2020): 10–15. 31535635 10.4103/1673-5374.264440 PMC6862393

[190] A. M. Fortress , E. D. Hamlett , E. M. Vazey , et al., “Designer Receptors Enhance Memory in a Mouse Model of Down Syndrome,” Journal of Neuroscience: The Official Journal of the Society for Neuroscience 35, no. 4 (2015): 1343–1353. 25632113 10.1523/JNEUROSCI.2658-14.2015 PMC4308587

